# Dysregulation of N-glycosylation by *Rpn1* knockout in spermatocytes induces male infertility via endoplasmic reticulum stress in mice

**DOI:** 10.7150/ijbs.106468

**Published:** 2025-03-03

**Authors:** Mingyu Zhang, Mengjing Li, Hanzhen Li, Yanling Wan, Shuang Yang, Shuhui Ji, Haobo Zhang, Chao Liu, Gang Lu, Xiaohua Jiang, Hongbin Liu

**Affiliations:** 1 Institute of Women, Children and Reproductive Health, Shandong University, 250012, China.; 2 State Key Laboratory of Reproductive Medicine and Offspring Health, Center for Reproductive Medicine, Institute of Women, Children and Reproductive Health, Shandong University, 250012, China.; 3 National Research Center for Assisted Reproductive Technology and Reproductive Genetics, Shandong University, Jinan, Shandong, 250012, China.; 4 Key Laboratory of Reproductive Endocrinoligy (Shandong University), Ministry of Education, Jinan, Shandong, 250012, China.; 5 Shandong Technology Innovation Center for Reproductive Health, Jinan, Shandong, 250012, China.; 6 Shandong Provincial Clinical Research Center for Reproductive Health, Jinan, Shandong, 250012, China.; 7 Shandong Key Laboratory of Reproductive Research and Birth Defect Prevention, Jinan, Shandong, 250012, China.; 8 Research Unit of Gametogenesis and Health of ART-Offspring, Chinese Academy of Medical Sciences (No.2021RU001), Jinan, Shandong, 250012, China.; 9 School of Basic Medical Sciences, Shandong University, Jinan 250012, China.; 10 State Key Laboratory of Medical Proteomics, Beijing Proteome Research Center, National Center for Protein Sciences (Beijing), Beijing Institute of Lifeomics, Beijing 102206, China.; 11 Center for Reproductive Medicine, the Second Hospital, Cheeloo College of Medicine, Shandong University, Jinan, Shandong 250012, China.; 12 Guangzhou Women and Children's Medical Center, Guangzhou Medical University, Guangzhou, 510623, China.; 13 CUHK-SDU Joint Laboratory on Reproductive Genetics, School of Biomedical Sciences, the Chinese University of Hong Kong, Hong Kong, China.; 14 Center for Reproduction and Genetics, Department of Obstetrics and Gynecology, The First Affiliated Hospital of USTC, Division of Life Sciences and Medicine, University of Science and Technology of China, Hefei, Anhui, 230001, China.

**Keywords:** RPN1, N-glycosylation, meiosis, endoplasmic reticulum stress, fertility

## Abstract

N-glycosylation protein modification plays a crucial regulatory role in numerous biological processes, although their contribution to male reproduction in mammals remains largely undefined. Here, we found that Ribophorin I (RPN1), a subunit of oligosaccharyltransferase complex, is indispensable for spermatogenesis in male germ cells. Germ cell-specific *Rpn1* knockout results in significant inhibition of the progression of meiosis, consequently disrupting homologous chromosome pairing, meiotic recombination, and DNA double strand breaks repair during meiosis. N-glycoproteomic profiling revealed that glycosylation levels are reduced in endoplasmic reticulum-associated proteins, while functional analyses showed that *Rpn1* deficiency could inhibit endoplasmic reticulum function and trigger endoplasmic reticulum stress during meiosis and increasing apoptosis levels in mice. These findings highlight the essential physiological functions of N-glycosylation modification in male spermatogenesis and expand our understanding of its role in male fertility.

## Introduction

Meiosis is a specialized form of cell division in which genetic material is duplicated once and cells divide twice in succession, resulting in gametes with half the number of chromosomes as somatic cells^1^. Meiotic prophase I involves the exchange of genetic materials between homologous chromosomes and comprises five distinct substages, including the leptotene, zygotene, pachytene, diplotene, and diakinesis. DNA double strand breaks (DSBs) trigger the recruitment of meiosis-related factors, thus initiating homologous recombination to repair damaged DNA. During the zygotene stage, a set of recombination proteins are recruited to meiotic DSBs, leading to the formation of recombination foci and synapsis of homologous chromosomes^2^. Subsequently, DMC1 and RAD51 mediate strand-invasion and promote the formation of a displacement loop^3^, ^4^. When the chromosomes are fully synapsed in the pachytene stage, the recombination foci continue to mature, and ultimately serve as the site of crossover events. Homologous chromosome pairing, synapsis, recombination and separation together constitute the key events of meiosis, and abnormalities in any of these stages will cause male azoospermia/oligospermia^5^.

N-glycosylation is important post-translational modifications (PTM) in a wide range of biological processes of eukaryotes. To catalyze this modification, oligomannose-type N-glycans are transferred as a structural unit to a specific asparagine-containing protein sequence motif (Asn-X-Thr/Ser, X≠Pro) in the endoplasmic reticulum (ER)^6^. Mutations leading to changes in the degree of glycosylation or the structure of the glycan chain have been found to often result in the occurrence of various diseases^7^, ^8^. For instance, abnormal polysaccharide synthesis and formation of glycoside-linked glycoproteins can lead to congenital disorders of glycosylation (CDGs)^9^. Both human congenital disorder of glycosylation type Ia (CDG-Ia) and mouse CDGs (e.g., *Mgat2^-/-^*) manifest as testicular atrophy, azoospermia, and sterility, suggesting that glycosylation plays an important role in spermatogenesis^10^, ^11^. Other mouse models with glycosylation defects, such as *Pmm2^-/-^* and *Alg3^-/-^*, exhibit similar reproductive phenotypes, further supporting the importance of glycosylation in male fertility^11^-^13^. The oligosaccharyltransferase (OST) complex is a key enzyme complex in the ER that mediates N-glycosylation, with most N-glycosylation events in eukaryotic cells dependent on its function. The OST complex consists of multiple subunits, including STT3A/B, ribophorin 1 (RPN1), and RPN2, which play essential roles in substrate recognition and glycan transfer^14^. Previous studies have shown that different subunits have distinct functions in N-glycosylation. For example, STT3A is primarily involved in co-translational glycosylation, while STT3B is closely associated with post-translational glycosylation^14^, ^15^. RPN1, a highly conserved subunit of the OST complex, mainly participates in N-glycosylation by binding specific glycoprotein substrates and promoting glycan transfer. Unlike the STT3 subunits, which directly catalyze glycan transfer, RPN1 acts as a bridge in substrate recognition^16^. Previous studies have reported that newly synthesized membrane proteins transiently associate with RPN1 after exiting the Sec61 translocation channel, which suggests that RPN1 could retain potential substrates close to the catalytic subunit of OST to increase N-glycosylation efficiency^17^. RPN1 plays a critical role in the process of N-glycosylation, and previous studies have shown that *Rpn1* knockout in breast cancer cells could induce ERS, subsequently promoting apoptosis^18^. Although the role of *Rpn1* in N-glycosylation is well established, to our knowledge, its function has not been examined in spermatogenesis through genetic manipulation.

The OST complex plays a pivotal role in the co-translational glycosylation of nascent proteins in the ER, a process essential for proper protein folding, stability, and overall cellular homeostasis. While the respective functions of OST subunits in different tissues have been extensively studied, much less is known about their specific role(s) in male germ cell development, particularly spermatogenesis. Although RPN1 was the primary focus of our current study, other OST subunits also play critical roles in cellular viability. Deficiency for these subunits can impair glycosylation and exacerbate ER stress, disrupting cellular function. For example, the key OST complex subunits, STT3A and STT3B, both provide essential functions to the protein glycosylation process, while the loss of either protein leads to impaired glycosylation, subsequently affecting cellular survival and function^19^. Additionally, other OST subunits, such as TMEM258 and TUSC3, also mediate vital roles in regulating ER stress and cell viability. For instance, the loss of TMEM258 has been shown to exacerbate ER stress and is linked to intestinal inflammation^20^, while the loss of TUSC3 reportedly accelerates the progression of prostate cancer, suggesting a role in tumor cell proliferation^21^. Notably, suppression of Dad1, another OST complex component, has been shown to induce cell death by weakening adhesion in cardiomyocytes, highlighting its function in maintaining cellular integrity^22^. These studies depict distinct functions of individual OST complex subunits in maintaining cellular homeostasis, particularly in the context of ER stress responses, whereas defects in OST complex subunits result in impaired glycosylation processes and exacerbated ER stress, negatively affecting cell function and survival.

In eukaryotic cells, the ER is an organelle that performs essential functions in intracellular protein synthesis, calcium ion homeostasis, and phospholipid synthesis^23^, ^24^. The majority of proteins secreted by cells first enter the ER for folding and assembly, and only properly assembled proteins can be transported from the ER to the extracellular space^25^. The protein folding process in the ER is highly sensitive to changes in environmental conditions, such as redox status, Ca^2+^ levels, or inflammatory stimuli, which can interfere with folding, leading to the accumulation of misfolded or unfolded proteins in the ER lumen, and ultimately inducing endoplasmic reticulum stress (ERS)^26^. Impaired glycosylation typically results in disrupted protein maturation and incorrect protein folding, which in turn activates the unfolded protein response (UPR) pathway in the ER^27^, which is composed of three ER-bound proteins: PKR-like ER kinase (PERK), activating transcription factor 6 (ATF6), and inositol-requiring enzyme 1 (IRE-1)^28^, ^29^. Upregulation of the UPR in response to ERS can also involve increased production of reactive oxygen species (ROS), and these pathways can form a feedback regulatory loop, reciprocally stimulating each other, and disrupting cellular homeostasis^30^. In some cases, the UPR can protect cells from stress-induced damage and restore intracellular homeostasis; however, prolonged activation of the UPR can serve as causal factor in many diseases^31^, ^32^. Several recent studies have shown that ERS is also a key pathogenic factor in abnormal spermatogenesis, and loss of function in proteins regulating ERS can impair spermatogenesis. The ER chaperone, HSPA5 (aliases GRP78 or BiP), plays a crucial role in suppressing ERS^33^, ^34^. *Hspa5* knockout in germ cells can impair proliferation and differentiation in spermatogonia, leading to dramatically reduced abundance of differentiated spermatogonia, disrupted meiosis, and resultant testicular atrophy and male infertility^35^. *Hspa2* deletion can also lead to abnormal synaptonemal complex assembly in the spermatocyte nucleus, resulting in meiotic failure and infertility^36^. Alternatively, *Atf6* deficiency leads to ERS in spermatocytes of male mice, downregulating *Tssk4* expression, which results in decreased fertility^37^. Although the ER is known to play a crucial role in reproductive biology, including spermatogenesis, the exact mechanisms underlying the specific functions of the ER in male reproduction, and the role of post-translational N-glycosylation modification in this mechanism, remain unclear.

In the current study, to define the precise function of RPN1 in male reproduction, we generated conditional knockout mice by ablating RPN1 specifically in pre-meiotic germ cells of* Stra8-GFPCre* mice. Examination of testes histology and structure in these mice showed a deficiency for spermatocytes due to the progression of meiotic prophase was delayed or blocked, which negatively affected DSBs repair and crossover formation. In addition, male mice were completely infertile. N-glycoproteomic analysis revealed that RPN1 deficiency specifically affects ER-associated glycoproteins during spermatogenesis, resulting in ERS. Overall, our study found that disruption of RPN1 leads to ERS, which in turn impairs meiosis, increases apoptosis, and ultimately results in abnormal spermatogenesis.

## Methods

### Mice

C57BL/6J mice were used to generate *Rpn1* conditional knockout models in this study. Mice with disrupted *Rpn1* in germ cells were created by crossing *Rpn1^flox/flox^* mice with *Vasa-Cre* and *Stra8-GFPCre* mice, respectively. *Stra8-GFPCre* mice were obtained from Dr. Minghan Tong's Laboratory at Chinese Academy of Sciences. All experimental protocols were approved by the Animal Ethics Committee of the School of Medicine, Shandong University (Jinan, Shandong Province, China). All experiments with mice were conducted following the Institutional Animal Care and use Committee protocols (IACUC; #2018-77) of Shandong University. All mice were maintained under specific pathogen-free (SPF) conditions with access to water and food and 12 h dark/12 h light cycles.

### Conditional knockout mice generation and genotyping

*Rpn1^flox/flox^* mice were generated by Cyagen Biosciences Inc. (Guangzhou, China) by gene editing with the CRISPR-Cas9 system. According to the structure of *Rpn1* gene, exon2 of the *Rpn1*-201 (ENSMUST00000032143.7) transcript was selected as target for deletion. Briefly, Cas9 mRNA and gRNA was transcribed* in vitro*. Vectors encoding Cas9, sgRNA, and donor were microinjected into the fertilized eggs of C57BL/6J mice. Zygotes were transplanted into female mice to obtain positive F0 mice, which were confirmed by PCR and Sanger sequencing. A stable F1 generation mice were obtained by mating positive F0 generation mice with wild-type C57BL/6J mice. Mice genotyping was conducted using genomic DNA extracted from mouse toes for PCR amplification. The flox and wild-type bands were 407 base pairs(bp) and 306bp, respectively. The *Rpn1* knockout band was 443bp. The primer sequences used for genotyping by PCR and PCR conditions are listed in Table S1.

### Spermatocyte spreads and immunofluorescence staining

Testicular cells from 1- to 2-month-old mice were prepared for meiotic chromosome spreads and subsequent immunofluorescence staining, as previously reported^38^. In brief, the tunica albuginea was removed after the testicles were washed in phosphate-buffered saline (PBS). Then, 1.5-2 cm seminiferous tubules were soaked in hypotonic extraction buffer (30 mM Tris, 50 mM sucrose, 17 mM trisodium citrate dihydrate, 5 mM ethylenediaminetetraacetic acid [EDTA], 0.5 mM DTT, and 0.5 mM PMSF, pH = 8.2) for 30-40 min at room temperature. The tubules were subsequently dispersed mechanically to release the spermatocytes into solution containing 100 mM sucrose (pH8.2) and spread on a clean glass slide with 1% paraformaldehyde (PFA; pH 9.2), containing 0.15% Triton X-100. The slides were air-dried in a humidity box for at least 2 hours prior to immunofluorescence staining.

For immunostaining, slides were placed into 1×PBS containing 5% bovine serum albumin (BSA) for 10 min, then blocked with 5% BSA for at least 1 hour at room temperature. Next, slides were incubated with primary antibodies (Table S2) at 4°C overnight. After incubation, slides were washed and incubated with secondary antibodies. The nuclei were stained with DAPI (Vector Laboratories) and sealed by Vectashield (Vector, H-1000, Shanghai, China).

### Histology, immunostaining and TUNEL analyses

Testes and ovaries extracted from mice were fixed with 4% PFA (Solarbio, Beijing, China, P1110) and Bouin's solution at 4°C overnight for histological analysis and immunostaining, respectively. The samples were dehydrated, embedded in paraffin, and cut into 5μm sections. The sections were stained with hematoxylin for histological analysis. For immunofluorescence analysis, sections were boiled in 10 mM sodium citrate buffer (pH 6.0) for 20 min, soaked in 1×PBS containing 5% BSA for 10min, and then blocked with 5% BSA for 1 hour in a humidity box. Sections were incubated with primary and secondary antibodies (Table S2). After a brief rinse with PBS, slides were counterstained by DAPI.

TUNEL assays were carried out strictly following the manufacturer's instructions accompanying the One Step TUNEL Apoptosis Assay Kit (Meilunbio, MA0223). Images were obtained with an LSM 780/710 microscope (Zeiss) or SP8 microscope (Leica).

### Western blotting

Testes were dissected and immediately suspended in NP40 buffer containing protease inhibitor cocktail (Roche, 04693116001). After sufficient oscillation and cracking, supernatant protein extracts were incubated at 95 °C with 1×SDS loading buffer (Beyotime, P0015L) for 5 min. Equal concentrations of protein were separated by 10% SDS-polyacrylamide gel electrophoresis (SDS-PAGE), then blotted onto polyvinylidene difluoride (PVDF) membranes (Millipore, USA). The membranes were blocked with 5% nonfat milk for 1 hour at room temperature and incubated with primary antibodies at 4 °C overnight. After washing, membranes were incubated with HRP-conjugated secondary antibodies, then photographed in a Bio-Rad ChemiDoc MP 453 imaging System, using Image Lab Software (Bio-Rad, USA) for image analysis. β-Actin was used as the loading control. The primary and secondary antibodies used for Western blotting are listed in Table S2.

### RNA extraction and quantitative reverse-transcription PCR (RT-qPCR)

FastPure Complex Tissue/Cell Total RNA Isolation Kit (Vazyme, RC113-01-AB) was used to extract total RNA from tissues and germ cells from various development stages and acquired ultimately cDNA according to the manufacturer's protocols(Yeasen,11156ES10). RT-qPCR was carried out on LightCycler@96 Real-Time PCR system (Roche) by using SYBR Green Premix Pro Taq HS qPCR Kit (AG, 11701). *β-Actin* and *Gapdh* were used as the housekeeping genes. The formula of 2^-ΔΔCt^ was used to calculate the relative gene expression. Table S1 lists the primers used in this experiment.

### Detection of glycoprotein with concanavalin A (Con A)

After SDS-PAGE of the testicular protein lysate, the gel was placed in Con A dilution buffer (100 μg/mL, Beyotime, ST2062) and incubated overnight at 4 °C. After washing, the gel was placed in Coomassie Brilliant Blue and incubated at room temperature for 10 minutes, then rinsed with running water until completely destained. Then photographed in a Bio-Rad ChemiDoc MP 453 imaging System, using Image Lab Software (Bio-Rad, USA) for image analysis.

### Enzymatic deglycosylation analysis of glycoprotein

To verify the glycosylation of proteins, PNGase F (Beyotime, P2318S) was employed following the manufacturer's instructions. Briefly, 10-100 μg of protein from the whole-cell lysates was mixed in a total reaction volume of 10 μL, including 1 μL of 10 × denaturing buffer, and then denatured by heating at 100°C for 10 minutes. After cooling to room temperature, 2 μL of Reaction Buffer (10×), 2 μL of Renaturing Buffer (10×), and 6 μL water were combined to make up a 20 μL reaction volume. 1 μL of PNGase F (500 U/μL) was added and thoroughly mixed, followed by incubation at 37 °C for 1-4 hours. Subsequently, the mixture was subjected to WB with the glycoprotein antibodies.

### Flow cytometry analysis of spermatogenic cells

Flow cytometry analysis of spermatogenic cells was performed as previously described^39^. Briefly, after removing the tunica albuginea, testes were dispersed lightly with tweezers and the seminiferous tubules were incubated in 1× collagenase type I (120 U/ml, Thermo Fisher Scientific, 17100017) at 35 °C with gentle rotation for 10 min. After further digestion in 5 mL of 0.25% trypsin containing 5 mg/mL DNase I for 7 min at 35 °C with rotation, tubule fragments were pipetted up and down to disperse germ cells. 0.5 mL of fetal bovine serum (FBS) was added to inactivate the trypsin and the suspension was filtered through 70 μm honeycomb filters. The extracts were centrifuged at 500 g for 5 min and the supernatant was discarded. Pellets were resuspended in DMEM containing 10% FBS. After staining with Hoechst 33342 (1x10^6^/ml, 3 μg/μL) at 35°C for 40 min, cells were sorted by flow cytometer (BD Biosciences, FACS Aria II, USA) according to different fluorescent channels. The germ cell samples were then used for ROS detection and Ca^2+^ detection following the instructions accompanying each respective kit.

### Detection of ROS levels

An ROS assay kit (Beyotime, S0033S) was used to assess ROS levels in testes. The DCFH-DA probe was diluted in DMEM medium to prepare culture droplets, which were subsequently incubated at 37°C for 30 min to achieve equilibrium. Spermatogonia at various stages were obtained through FACS and placed into droplets containing 10 µM DCFH-DA. Droplets were incubated at 37°C for 30 minutes in darkness. After washing, analysis was performed using a FACS Aria II flow cytometer (BD Biosciences, USA).

### Proteomics and N-glycoproteomic analysis

PD12 testicular samples from WT and *Rpn1*-SKO mice were subjected to proteomics and N-glycoproteomic analysis using the same samples. After three rinses with PBS, the samples were placed in micro centrifuge tubes, flash frozen in liquid nitrogen. The samples were transported on dry ice to Beijing Proteome Research Center for proteomics, N-Glycoproteomic, and corresponding data analyses.

For total proteomics, samples were ground into cell powder with liquid nitrogen and lysed in a buffer containing 8 M urea and 1% protease inhibitor cocktail, followed by sonication on ice. After centrifugation at 12,000 g for 10 min at 4 °C, supernatants were collected, and protein concentration was determined using a BCA kit. Proteins were precipitated with 20% (m/v) TCA, washed with cold acetone, dried, and redissolved in 200 mM TEAB. Trypsin digestion (1:50 ratio) was performed overnight, with reduction by DTT and alkylation by IAA. Finally, LC-MS analysis was conducted.

For N-Glycoproteomic analysis, the samples were modified and enriched after lysis and trypsin digestion. Briefly, dissolve the peptide in 200 μL of enrichment buffer (80% acetonitrile/5% trifluoroacetic acid), transfer the supernatant to a HILIC microcolumn, and centrifuge at 1000 g for 15 minutes. Wash the column three times with enrichment buffer, then elute glycopeptides using 0.1% trifluoroacetic acid, 50 mM ammonium bicarbonate, and 50% acetonitrile. Combine eluates, vacuum dry, reconstitute in 50 μL of 50 mM ammonium bicarbonate (prepared with heavy water), add 2 μL PNGase F, and digest overnight at 37 °C. Desalt using C18 ZipTips, dry, and prepare for LC-MS analysis. Thresholds of *p* value < 0.05 and |fold change| >1.5 were used to identify significant differentially regulated proteins in comparisons between WT and *Rpn1*-SKO samples.

Gene Ontology (GO) functional annotation analysis with Fisher's exact test to determine significantly enriched terms (*p* value < 0.05) among total identified proteins. The Kyoto Encyclopedia of Genes and Genomes (KEGG) database was utilized to conduct pathway enrichment analysis, with Fisher's exact test (*p* value < 0.05) to assess significant, differentially enriched pathways from among total detected proteins. The R package clusterProfiler and Metascape (http://metascape.org) were used for GO and KEGG pathways enrichment analyses.

### Statistical analysis

Unless otherwise stated, all statistical analyses of experimental data in this study were conducted using at least three independent repetitions. Two-tailed unpaired Student's t-tests were used to identify significant different differences between groups (with significant differences at *p < 0.05, and highly significant differences at **p < 0.01). All data are presented as means ± SEM. Statistical analyses were conducted using GraphPad Prism 8 software (GraphPad Software, San Diego, CA, USA).

## Results

### RPN1 is highly expressed in pachytene/diplotene spermatocytes in mice

**Figure 1:**
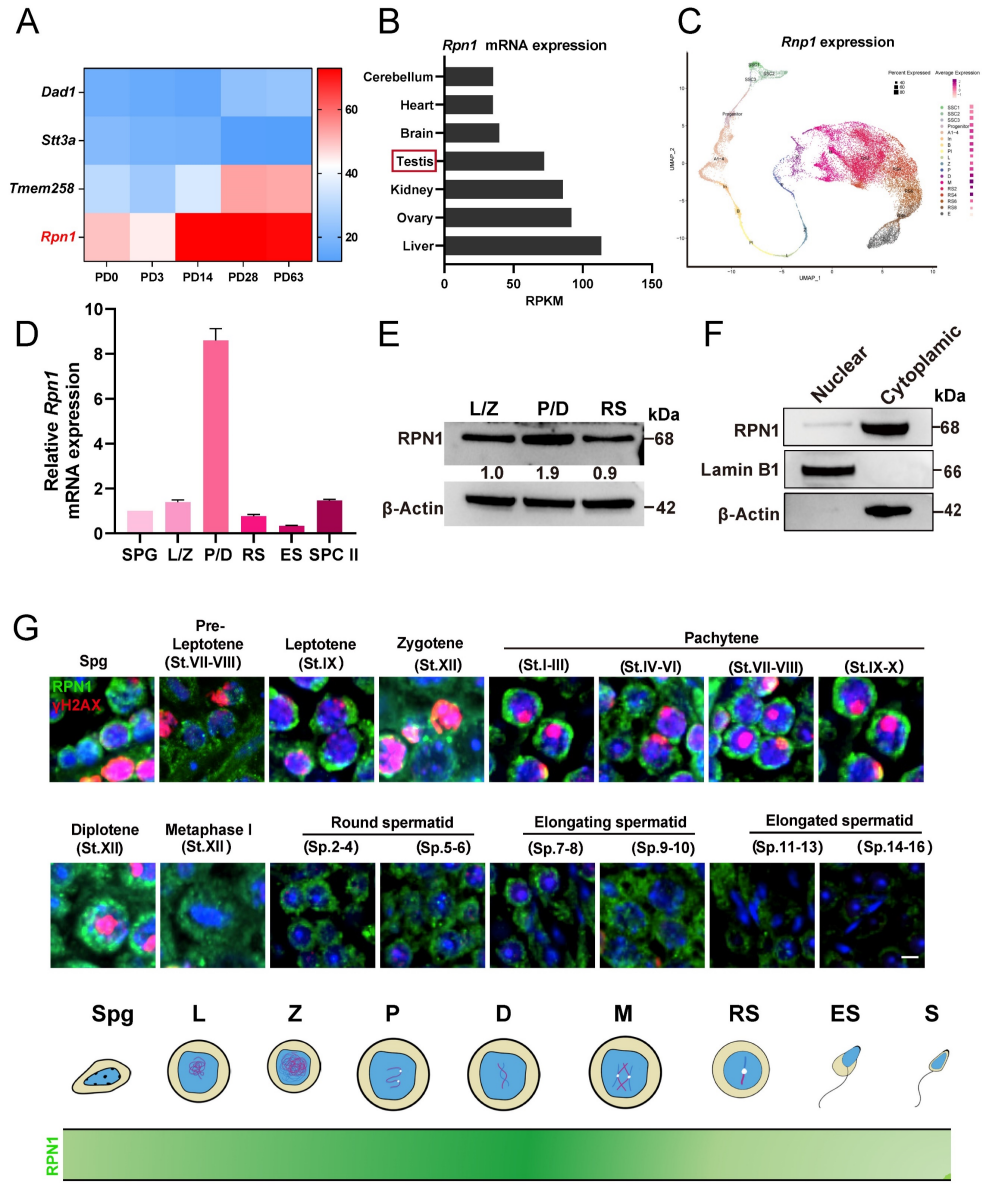
** The expression and localization of RPN1 in testes. A.** Heatmap analysis of OST subunit expression at various postnatal stages (P0, P3, PD14, PD28, and PD63) in mouse testes, based on data from the Evo-devo mammalian organ database (http://apps.Kaessmannlab.org/evodevoapp/). **B.** The analysis of *Rpn1* expression in different tissues using the Evo-devo mammalian organ database (http://apps.Kaessmannlab.org/evodevoapp/). **C.** UMAP map showing the expression of *Rpn1* in different mouse germ cells using the Male Health Atlas (http://malehealthatlas.cn/). SSC1-3: Spermatogonial stem cells I-III; Progenitor: spermatogonia progenitor cell; A1-4: A1-4 spermatogonia; In: Intermediate spermatogonia; B: Type B spermatogonia; PI: Preleptotene; L: Leptotene; Z: Zygotene; P: pachytene; D: Diplotene; M: Metaphase; RS: Round spermatid. E: Elongating spermatid. **D.** RT-qPCR detection *Rpn1* in spermatogenic cells isolated from adult WT mice using FACS. SPC II: Secondary spermatocytes; *β-Actin* served as loading control. **E.** WB detection of RPN1 in spermatogenic cells isolated from adult WT mice using FACS. The relative expression level of the RPN1 protein was calculated by quantifying the gray value of RPN1/β-Actin for each sample. **F.** WB analysis of RPN1 in the nucleus and cytoplasm of spermatogenic cells from 2M WT testes. β-Actin and Lamin B1 were used to indicate the separation efficiency of nuclear and cytoplasmic proteins, respectively.** G.** Spatiotemporal expression pattern of RPN1 in postnatal male germ cells. DNA was stained with DAPI. Scale bar, 5 μm.

To explore the role of *Rpn1* in spermatogenesis, we examined its expression using the Evo-devo mammalian organ database^40^. The mRNA expression level of *Rpn1* increased sharply after postnatal day 14 (PD14) and then remained at a high level (Figure 1A). Further monitoring of RPN1 levels by Western blot (WB) in the testes of C57BL/6J mice from PD6 through 3-month-old (3M) revealed that RPN1 protein abundance increased sharply during PD12 to PD18 (Figure S1A and S1B). We next analyzed *Rpn1* expression in different tissues using this database and found that high *Rpn1* expression were observed in both testes and ovaries (Figure 1B). The RPN1 expression pattern was further confirmed by WB and reverse-transcription quantitative real-time PCR (RT-qPCR) (Figure S1C and S1D). We subsequently analyzed *Rpn1* at various stages of germ cell development using single-cell sequencing data of male-specific tissues and organs in mice and humans^41^. The results showed that *Rpn1* was expressed throughout germ cells, especially in spermatocytes during meiosis (Figure 1C).

We then isolated germ cells at various developmental stages from wild-type (WT) adult mice (2M) by fluorescence-activated cell sorting (FACS), including spermatogonia (SPG), spermatocytes at leptotene/zygotene (L/Z) and pachytene/diplotene (P/D), round spermatids (RS), elongated sperms (ES) and secondary spermatocytes (SPC II). After verifying the purity of the sorted cells (Figure S1E), we performed RT-qPCR and WB to assess RPN1 expression levels. *Rpn1* expression was detectable in spermatogenic cells entering early meiotic prophase I (L/Z), and increased sharply in P/D spermatocytes, then declined to relatively lower levels in the RS stage (Figure 1D). The RPN1 expression pattern was further confirmed by WB (Figure 1E). We then fractionated testes extracts from adult mice for WB analysis and co-staining with the cytoplasmic and nuclear markers, β-Actin and Lamin B1, respectively, indicated that RPN1 was present in the cytoplasmic fraction (Figure 1F). In addition, we evaluated the subcellular localization of RPN1 in WT adult testes by immunofluorescence (IF) staining with antibodies targeting RPN1 and γH2AX (which indicate various spermatocyte types). The results showed that RPN1 was detected in spermatogonia and leptotene through zygotene spermatocytes, but at much higher levels in pachytene and diplotene spermatocytes (Figure 1G). These findings thus implied that RPN1 could potentially play a role in spermatogenesis in mice.

### Germ cell-specific deletion of *Rpn1* results in male infertility

To futher study the *in vivo* function of RPN1 in the reproductive system, we initially created a global *Rpn1* knockout mouse model (referred to as *Rpn1*-gKO) by mating germline Cre-deleter mice with *Rpn1*-floxed mice (Figure S2A). However, no *Rpn1^-/-^* pups were obtained from this crossing, as detected from DNA genotyping. We then crossed *Vasa-Cre* transgenic mice with the *Rpn1^flox/flox^* mice to obtain *Rpn1^flox/-^; Vasa-Cre* (referred to as *Rpn1*-VKO, hereafter) mice, in which *Rpn1* was specifically inactivated at embryonic day 15.5 (Figure S2B). Gross morphological observations revealed that the testes of *Rpn1*-VKO mice were significantly smaller and that the testes-to-body weight ratio was significantly decreased in *Rpn1*-VKO mice compared with WT controls (*Rpn1^flox/+^* and *Rpn1^flox/flox^* male littermate; Figure S2C and S2D). Histological examination by hematoxylin and IF staining showed that the seminiferous tubules of *Rpn1*-VKO mice were almost completely devoid of germ cells (Figure S2E and S2F), suggesting that RPN1 is required for spermatogenesis in mice. These results hinder us from investigating the physiological functions of RPN1 in the reproductive system.

**Figure 2:**
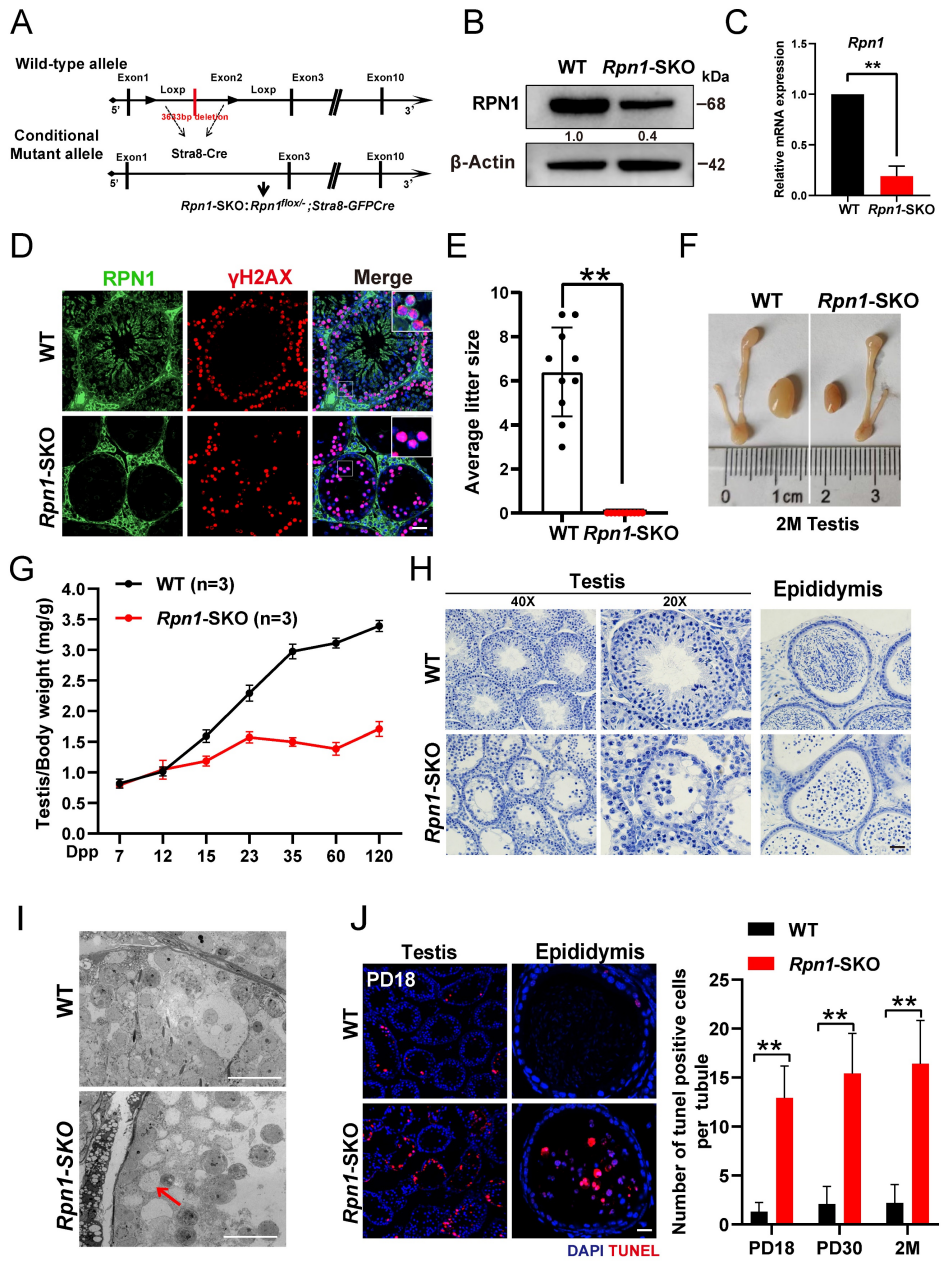
** Essential role of RPN1 in spermatogenesis and male fertility in mice. A.** Schematic for *Rpn1* conditional knockout strategy in germ cells. **B.** WB analysis of RPN1 knockout efficiency in testes of adult WT and *Rpn1*-SKO mice. β-Actin served as loading control. **C.** RT-qPCR analysis of *Rpn1* knockout efficiency in testes of adult WT and *Rpn1*-SKO mice. *β-Actin* served as loading control. **D.** Immunofluorescence staining for RPN1 in WT and *Rpn1*-SKO testes. γH2AX was co-stained to indicate spermatocytes. Scale bars, 30 μm. **E.** Breeding assays of *Rpn1*-SKO male mice and age-matched WT female mice for 6 months. Cumulative number of pups per male mating with one female mouse. Data represent means ± SEM. **p < 0.01 by Student's t-test. **F.** Morphology of testes derived from WT and *Rpn1*-SKO mice at 2M. **G.** Testis weight/body weight ratios in WT and *Rpn1*-SKO mice. The numbers of analyzed mice are indicated (n). Data represent means ± SEM. **p < 0.01 by Student's t-test. **H.** Histological staining of testes and epididymis from WT and *Rpn1*-SKO mice. Scale bars, 20 μm. **I.** TEM imaging of seminiferous epithelium in testicular sections from 2M WT and *Rpn1*-SKO mice. Scale bar, 5 µm. **J.** Left: TUNEL assays of testis sections from PD18 WT and *Rpn1*-SKO mice. Scale bar, 30 µm. Right: Quantification of TUNEL^+^ cells per tubule of different ages. Data represent means ± SEM, **p < 0.01 by Student's t-test.

We then further investigated the role of *Rpn1* in meiosis by crossing *Rpn1^flox/flox^* mice with *Stra8-GFPCre* transgenic mice, which express Cre recombinase in male germ cells from PD3, to obtain *Rpn1^flox/-^*; *Stra8-GFPCre* mice (*Rpn1*-SKO; Figure 2A). WB, RT-qPCR and IF staining confirmed that both RPN1 protein and mRNA expression levels were significant decrease in *Rpn1*-SKO testes compared with that of WT controls (Figure 2B-2D). Fertility tests for 6 months showed that *Rpn1*-SKO male mice were completely infertile, although body weight was comparable between *Rpn1*-SKO and WT mice (Figure 2E). We also noted that the testes of *Rpn1*-SKO male mice were much smaller than those of WT males, with significantly decreased testes weights and testes-weight-to-body-weight ratios (Figure 2F and 2G, Figure S2G). Histological analysis showed that *Rpn1*-SKO testes had very few spermatocytes and contained apoptotic cells or were almost empty, whereas the seminiferous tubules of WT mice contained abundant spermatocytes at various stages (Figure 2H). Additionally, we found that a small number of RS were present in the seminiferous tubules of *Rpn1*-SKO mice. We used fluorescein-conjugated peanut agglutinin (PNA), a protein that specifically binds to the outer acrosomal membrane, and found that only about 15% of the seminiferous tubules contained RS, and these RS could develop at most up to Golgi phase (steps 1-3, Figure S2H-2J). Furthermore, transmission electron microscopy (TEM) confirmed the defective development of germ cells within the seminiferous tubules of *Rpn1*-SKO mice. The WT epithelium was characterized by tightly packed layers of developing germ cells. In contrast, the *Rpn1*-SKO epithelium displayed a highly disorganized structure with reduced cell numbers of only loosely attached or completely detached germ cells (Red arrow, Figure 2I). The number of apoptotic cells in *Rpn1*-SKO testes were significantly increased by TUNEL assay even at the early PD18 stage, and the number of TUNEL-positive cells further increased with age (Figure 2J). These results suggest that the development of spermatocytes may have a defect during the first wave of spermatogenesis. No sperm were found in the epididymis of adult *Rpn1*-SKO mice, and there were also a large number of apoptotic cells present (Figure 2H and 2J). Collectively, these results indicated that deficiency of RPN1 in male germ cells caused abnormal spermatogenesis with substantial levels of apoptosis, ultimately leading to male infertility.

### RPN1 deficiency results in severe defects in meiosis during spermatogenesis in mice

**Figure 3:**
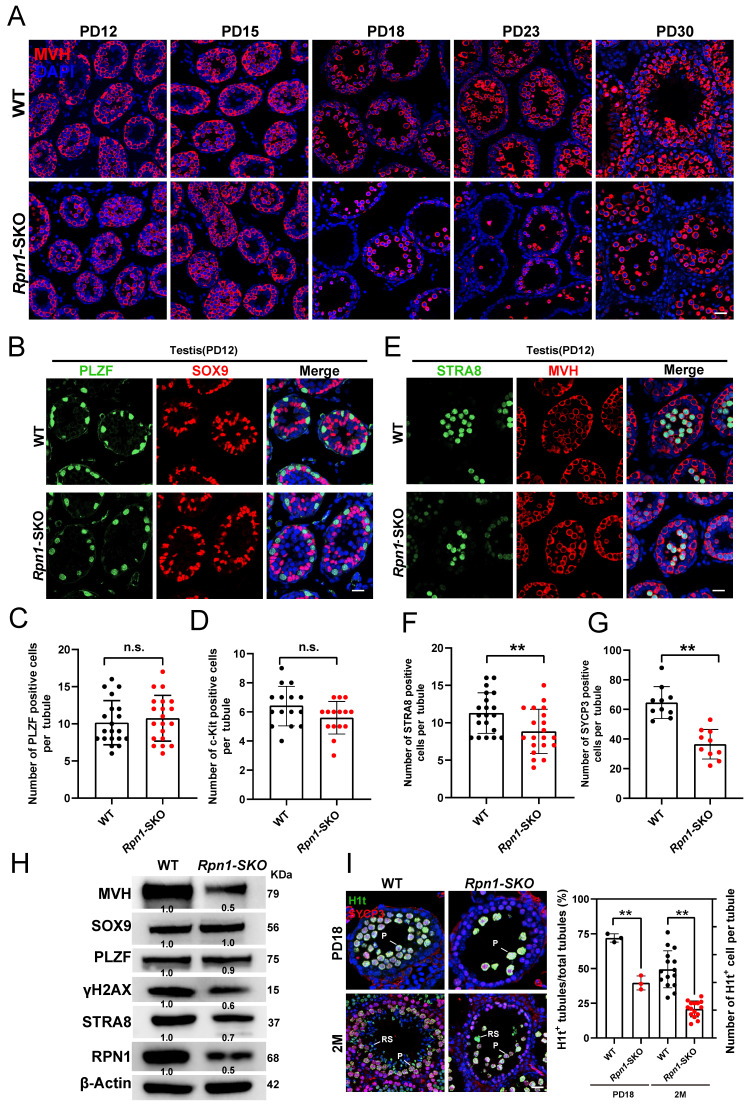
** Defects in spermatogenesis of *Rpn1*-SKO mice. A.** IF staining for MVH in testes of WT and *Rpn1*-SKO mice from PD12 to PD30. Scale bar, 30 µm. **B.** Immunofluorescence (IF) co-staining for PLZF (green) and SOX9 (red) in WT and *Rpn1*-SKO mice at PD12. Scale bar, 30 µm. **C.** Quantification of PLZF^+^ cells per tubule in PD12 WT and *Rpn1*-SKO mice. Data represent means ± SEM. n.s., not significant. **D.** Quantification of c-KIT^+^ cells per tubule in PD12 WT and *Rpn1*-SKO mice. Data represent means ± SEM. n.s., not significant.** E**. IF co-staining for STRA8 (green) and MVH (red) in WT and *Rpn1*-SKO mice at PD12. Scale bar, 30 µm.** F.** The quantification of STRA8^+^ cells per tubule in WT and *Rpn1*-SKO mice at PD12. Data represent means ± SEM. **p < 0.01 by Student's t-test.** G.** The quantification of SYCP3^+^ cells per tubule in WT and *Rpn1*-SKO mice at 2M. Data represent means ± SEM. **p < 0.01 by Student's t-test. **H.** WB detection of key protein levels in adult WT and *Rpn1*-SKO testes. β-Actin served as loading control. **I.** Left: IF co-staining for H1t (green) and SYCP3 (red) in WT and *Rpn1*-SKO mice at PD18 and 2M. Scale bar, 30 µm. Right: Number of seminiferous tubules containing H1t^+^ cells per total tubules. Data represent means ± SEM. **p < 0.01 by Student's t-test.

Given the defects in fertility and spermatogenesis observed in *Rpn1*-SKO mice, we next explored the developmental stage at which germ cell numbers initially decline by sampling testes from WT and *Rpn1*-SKO mice at PD12, PD15, PD18, PD23 and PD30 for IF and histology analysis. While no obvious difference was observed between *Rpn1*-SKO and WT testes at PD12, the number of germ cells was lower in *Rpn1*-SKO samples at PD15, and further sharply decreased from PD18 to PD30 (Figure 3A and Figure S3A). To verify whether depletion of RPN1 affects spermatogonial differentiation, we performed co-staining with IF antibodies targeting the undifferentiated spermatogonia marker, PLZF, and SOX9, to label Sertoli cells. Image analysis showed no difference in the abundance of stained cells between *Rpn1*-SKO and WT mice (Figure 3B and 3C and Figure S3B), which led us to focus next on stages of spermatogonial differentiation using the differentiated spermatogonia marker, c-KIT (Figure S3C). Image analysis again showed no significant difference between *Rpn1*-SKO and WT testes at age of PD12 (Figure 3D), indicating that RPN1 knockout in germ cells did not affect the differentiation of spermatogonial cells.

As spermatogonia develop into primary spermatocytes, which then enter meiosis^42^. We next explored whether RPN1 depletion affects meiotic entry by co-staining paraffin-embedded sections with fluorescence-labeled antibodies for MVH and either the meiosis initiation marker, STRA8, or spermatocyte meiosis marker, SYCP3 (Figure 3E and Figure S3D). We found that *Rpn1*-SKO testes harbored significantly fewer STRA8^+^ and SYCP3^+^ cells per seminiferous tubule compared to WT testes (Figure 3F and 3G), suggesting that RPN1 could participate in spermatocytes entry into meiosis. We also detected these markers in adult testes, which showed similar results in that the undifferentiated spermatogonial pools were unaffected. However, MVH, STRA8 and SYCP3 proteins were significantly reduced after RPN1 deletion (Figure 3H). We then examined expression of the testis-specific histone marker of the pachytene stage, H1t, which is upregulated in spermatocytes from mid- to late pachytene and diplotene to investigate the fate of *Rpn1*-SKO spermatocytes^38^. Analysis of H1t staining images revealed that *Rpn1*-SKO spermatocytes could reach at least the middle to late pachytene stage (i.e., PD18), whereas the first wave of spermatogenesis in WT mice reached the late pachytene stage. In addition, we observed that compared to the WT controls, the number of H1t^+^ cells in *Rpn1*-SKO testes was significantly reduced at both PD18 and 2M, suggesting that the progression of meiotic prophase was delayed or blocked in *Rpn1*-SKO spermatocytes (Figure 3I). These cumulative results indicated that RPN1 deletion leads to defects in meiosis during spermatogenesis in mice.

### *Rpn1* deficiency results in defects during synapsis and crossover recombination

**Figure 4:**
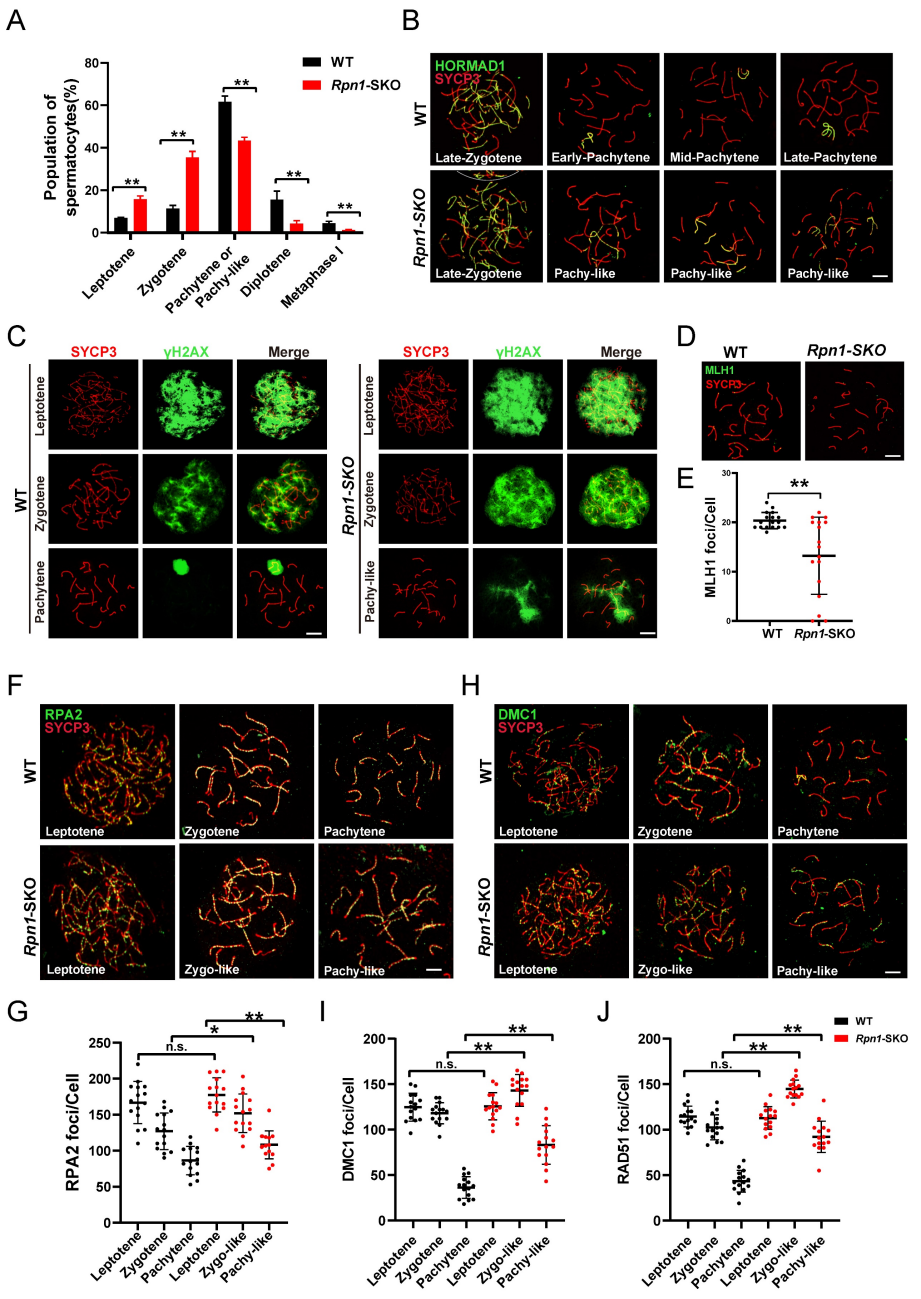
** Zygotene arrest with defects in synapsis and DSBs repair in *Rpn1*-deficient spermatocytes. A.** Percentage of meiotic spermatocytes in each stage of meiotic prophase. Data represent means ± SEM. **p < 0.01 by Student's t-test. **B.** Co-immunofluorescence staining of HORMAD1 (Green) and SYCP3 (Red) on surface-spread spermatocytes from the WT and *Rpn1*-SKO mouse testes. The dashed line separates the target cell from the adjacent unrelated cell. Scale bar, 5 µm. **C.** Representative images of WT and *Rpn1*-SKO spermatocytes at different stages of meiotic prophase co-stained for SYCP3 (red) and γH2AX (green). Scale bar, 5 µm. **D.** IF co-staining for SYCP3 (red) and MLH1 (green) in WT and *Rpn1*-SKO mice. Scale bar, 5 µm. **E.** Quantification of MLH1 foci per nucleus in WT and *Rpn1*-SKO mice from D. Data represent means ± SEM, **p < 0.01 by Student's t-test. **F.** IF co-staining for SYCP3 (red) and RPA2 (green) in WT and *Rpn1*-SKO mice. Scale bar, 5 µm. **G.** The quantification of RPA2 foci per nucleus in WT and *Rpn1*-SKO mice. Data represent means ± SEM, **p < 0.01 by Student's t-test. **H.** IF co-staining for SYCP3 (red) and DMC1 (green) in WT and *Rpn1*-SKO mice. Scale bar, 5 µm.** I.** Quantification of DMC1 foci per nucleus in WT and *Rpn1*-SKO mice from H. Data represent means ± SEM, **p < 0.01 by Student's t-test. **J.** Quantification of RAD51 foci per nucleus of WT and *Rpn1*-SKO. Data represent means ± SEM, **p < 0.01 by Student's t-test.

In the above study, we observed that *Rpn1*-SKO mice contained spermatocytes that could enter meiosis. To better understand the relationship between loss of *Rpn1* and the defects in meiosis observed in *Rpn1*-SKO spermatocytes, we performed IF staining for synaptonemal complex central element (SYCP1) and axial/lateral element (SYCP3) in chromosome spreads of spermatocytes from 1M mice^43^. All meiotic prophase I stages from leptotene to diakinesis could be detected in both *Rpn1*-SKO and WT testes (Figure S4A). The statistical analysis of the meiotic prophase population indicated that *Rpn1*-SKO mice had significantly fewer pachytene and diplotene cells, while zygotene cells were significantly increased compared with WT controls (Figure 4A), which was also reflected in subsequent assessment of spermatocyte clusters by FACS (Figure S4B and S4C). In addition, we observed that synapsis was completed on the autosomal axes in pachytene spermatocytes of WT mice (abnormal synapsis:5.8±0.7535%), whereas a large number of pachytene spermatocytes in *Rpn1*-SKO mice exhibited synapsis abnormalities (abnormal synapsis: 61.43±3.475%). Further analysis revealed a diversity synapsis defects in chromosome spreads of pachytene-like (pachy-like) *Rpn1*-SKO spermatocytes, such as multiple partners synapsed with different regions of a single chromosome, resulting in frequent pairing between non-homologous chromosomes (57.97±2.038%), or the presence of non-synaptic homologous chromosomes (38.40±1.415%; Figure S4D). In pachytene stage, HORMAD1 was removed from the autosomal axes and retained only in the unpaired regions of the XY body, whereas *Rpn1*-SKO spermatocytes retained HORMAD1 signals in several incomplete synaptic chromosomes (Figure 4B). These results suggested that meiosis might be suppressed or delayed during the zygotene-pachytene transition in the absence of RPN1, resulting in a small subset of spermatocytes with fewer defects that could progress into diplotene.

The completion of synapsis during the pachytene stage occurs concomitantly with completion of DSBs repair in autosomes^44^. Considering the defects in synapsis we observed in *Rpn1*-SKO testes, we hypothesized that DSBs repair might also be compromised. To test this possibility, we therefore examined DSBs formation and repair events by co-staining for the DNA DSBs marker, γH2AX, and SYCP3 (Figure 4C). We found that γH2AX signal had comparable intensity at the leptotene stage between *Rpn1*-SKO and WT, indicating that DSBs formation occurred normally in *Rpn1*-SKO spermatocytes. However, the strong γH2AX signals persisted on autosomes of pachy-like *Rpn1*-SKO spermatocytes, whereas they were restricted to the XY body in pachytene stage WT spermatocytes. These results suggested that DSBs repair was indeed defective in *Rpn1*-SKO spermatocytes. Further tests to determine whether these pachy-like spermatocytes could complete homologous recombination and form crossovers by co-staining for SYCP3 and crossover marker, MLH1, showed that MLH1 foci were obviously decreased in pachy-like spermatocytes (Figure 4D and 4E). These results indicated that RPN1 deletion causes severe defects in crossover formation.

It has been reported that the recombination protein MSH4 plays a role in stabilizing DNA strand exchange intermediates during homologous recombination, many of which eventually become crossovers, typically marked by MLH1. Next, we performed MSH4 immunostaining to examine how RPN1 deletion impacts homologous recombination intermediates during prophase I of meiosis. An increased number of MSH4 foci was observed in *Rpn1*-SKO spermatocytes compared to WT throughout the early to late pachytene stages (Figure S4E and S4F). Taken together, these results suggest that RPN1 performs crucial functions in processes related to DSBs repair and crossover formation in mouse spermatocytes.

To further investigate RPN1 function in DSBs repair and recombination during meiosis, we conducted IF staining for RPA2, a component of the RPA complex (which binds single strand 3' DNA overhangs on DSBs), as well as DMC1 and RAD51, key recombinases recruited after the RPA complex that mediate single strand invasion of the sister chromatid^3^, ^45^. We found that *Rpn1*-SKO and WT spermatocytes harbored a comparable number of RPA2 foci at the leptotene stage. With the progression of recombination in meiosis, RPA2 foci decreased at the zygotene and pachytene stages in WT spermatocytes. However, RPA2 foci were found to persist in zygotene-like (zygo-like) and pachy-like spermatocytes of *Rpn1*-SKO mice, indicating that RPA2 was not efficiently cleared (Figure 4F and 4G). We subsequently examined DMC1 and RAD51 foci to assess the effects of RPN1 deficiency on early homologous recombination. The results showed that foci of both of these recombinases decreased upon transition from the leptotene to pachytene stages in WT spermatocytes, indicating the successful repair of DSBs. However, these foci remained abundant at relatively high levels in *Rpn1*-SKO spermatocytes (Figure 4H-4J and Figure S4G). Overall, these results suggested that DSBs repair was likely disrupted by RPN1 depletion.

### N-glycoproteomics profiling shows reduced glycosylation levels in *Rpn1*-SKO testes

Considering the known role of RPN1 in facilitating N-glycosylation^46^, ^47^, we performed TMT-based quantitative proteomics and N-glycoproteomics analyses to quantify differences in proteins and N-glycosylation in PD12 testes between WT and *Rpn1*-SKO mice (i.e., the time point when meiosis I advances to the zygotene stage)^48^, ^49^. The PD12 time point was chosen because it precedes the onset of major meiotic defects, allowing us to investigate the underlying causes of meiotic failure. We first performed Gene Ontology (GO) and KEGG pathway enrichment analysis to identify significant differentially expressed proteins (DEPs) associated with *Rpn1*-SKO, which revealed that down-regulated proteins were enriched in biological processes related to “protein N-linked glycosylation” (Figure S5A). Consistent with this GO annotation, KEGG analysis showed enrichment with down-regulated DEPs in pathways involved in “various types of N-glycan biosynthesis” (Figure S5B). We then conducted lectin-binding assays using concanavalin A (Con A), which selectively binds to Asn-linked glycans to identify target proteins carrying N-GlcNAc modifications, to detect changes in N-glycosylation in the testes of *Rpn1*-SKO mice. Our results showed that lectin enrichment was significantly reduced in *Rpn1*-SKO testes compared to WT (Figure S5C), suggesting that RPN1 deletion resulted in severe defects in glycosylation. Additional WB assays verified the downregulation of some key DEPs identified by quantitative proteomics (Figure S5D).

**Figure 5:**
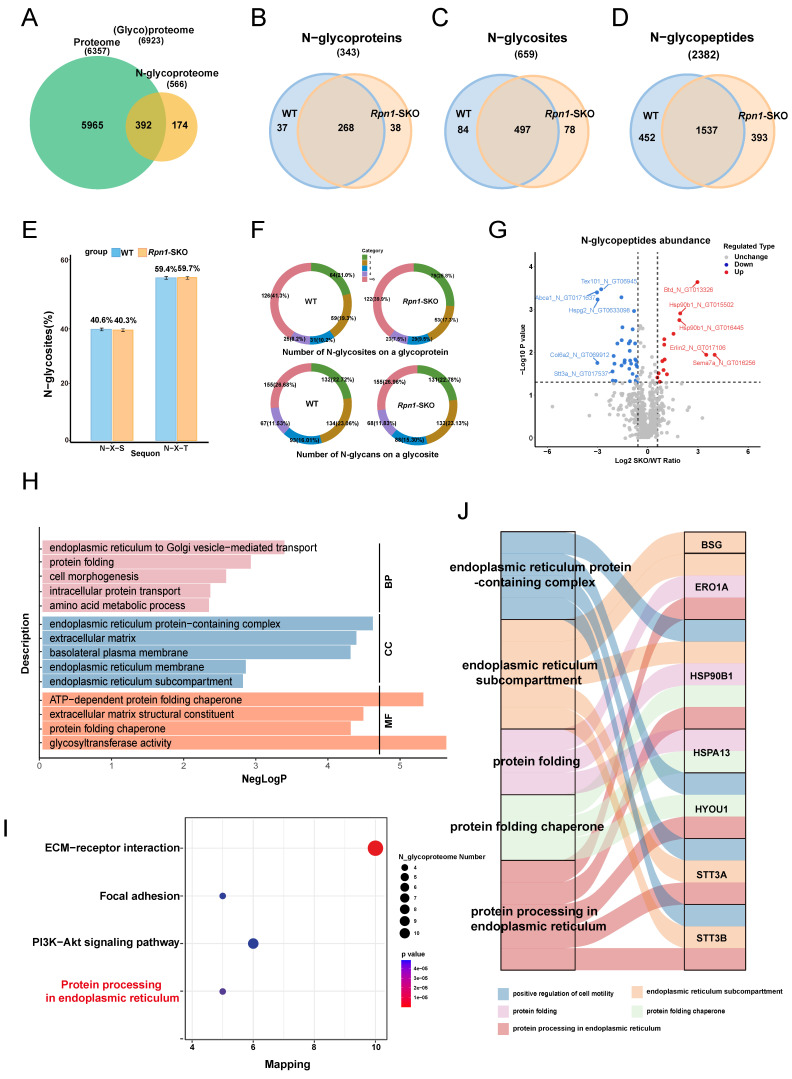
** Quantitative N-glycoproteomics analysis of WT and *Rpn1*-SKO testes. A.** Venn diagram of proteins identified in testes N-glycoproteome versus proteome. **B-D.** Venn diagrams of N-glycoproteins (B), N-glycosites (C), and N-glycopeptides (D) identified in PD12 WT and *Rpn1*-SKO testes. **E.** Percentage of N-glycopeptides with N-X-S/T tripeptide sequons in WT and *Rpn1*-SKO testes. **F.** Pie charts depict the percentage of N-glycoproteins with the identified number of N-glycosites per protein (upper) and the percentage of N-glycosites with the identified number of N-glycans per glycosite in WT and *Rpn1*-SKO testes (below). **G.** Volcano plot showing the number of differentially expressed glycoproteins in WT and *Rpn1*-SKO testes after normalizing to the total proteome. **H.** GO analysis of significantly downregulated glycoproteins. **I.** The bubble chart demonstrating KEGG pathway enrichment results of downregulated glycoproteins. **J.** Sankey diagram showing the connection between the interested GO enrichment terms with the relevant differentially expressed glycoproteins.

To assess whether the changes in N-glycopeptide abundance we observed in *Rpn1*-SKO testes resulted from altered protein expression or changes in N-glycosylation site occupancy, we normalized the variation in N-glycopeptide abundance with the abundance of corresponding proteins^50^. This pairwise comparison of protein levels between N-glycoproteomic and proteomic data showed that differences in the abundance of most N-glycopeptides were not due to changes in protein expression (Figure S5E). Overall, N-glycoproteome profiling identified 566 total proteins (including 343 N-glycoproteins), 659 N-glycosites, and 2382 N-glycopeptides in mouse testes (Figure 5A-5D). Among the identified proteins, 174 were only detected by N-glycoproteomic methods, while 392 were also quantified by proteomics (Figure 5A). Between WT and *Rpn1*-SKO testes, N-glycoproteomics analysis respectively identified 581 and 575 glycosites that mapped to 1989 and 1930 glycopeptides and 305 and 306 glycoproteins (Figure 5B-5D). The canonical N-glycosylation motif, N-X-S/T, was not affected by RPN1 deletion (Figure 5E). In WT testes, approximately 21% of the N-glycoproteins carried a single N-glycosylation site, 19.3% had two N-glycosites, 10.2% had three N-glycosites, 8.2% had four N-glycosites, and 41.3% had five or more N-glycosites. By contrast, approximately 25.8% of glycoproteins had one N-glycosite in *Rpn1*-SKO testes, while the percentages of proteins with two or more N-glycosites all decreased (Figure 5F, upper). In addition, no significant difference in glycan levels at each glycosite was observed between WT and *Rpn1*-SKO testes (Figure 5F, below).

We then conducted a differential glycopeptide abundance analysis and identified 50 N-glycopeptides that showed significant changes in the *Rpn1*-SKO group compared to controls (|FC| >1.5; *p* value< 0.05), including 15 glycopeptides from 10 glycoproteins with increased glycopeptide abundance and 35 glycopeptides from 27 glycoproteins with decreased glycopeptide abundance in *Rpn1*-SKO (Figure 5G and Table S3). GO analysis of the glycoproteins with decreased glycosylation in *Rpn1*-SKO testes showed enrichment in biological processes such as “endoplasmic reticulum to Golgi vesicle-mediated transport”, “protein folding”, “cell morphogenesis”, “intracellular protein transport”, and “amino acid metabolic process” (Figure 5H), while the “endoplasmic reticulum protein-containing complex” was the notably differentially expressed cellular component (Figure 5H). As the ER serves as the primary site for N-glycoprotein biosynthesis, folding, and posttranslational modification^26^. We subsequently focused on the role of these glycoproteins with decreased glycosylation in the ER. KEGG pathway analysis revealed significant enrichment in pathways associated with “Protein processing in endoplasmic reticulum” (Figure 5I). We subsequently performed the same GO analysis for up-glycosylated proteins to confirm whether the previous analysis results were specifically related to down-glycosylated proteins. We found that glycoproteins with increased glycosylation in *Rpn1*-SKO testes were enriched in biological processes such as the “ubiquitin-dependent ERAD pathway”, “glycosaminoglycan biosynthetic process”, “protein modification process”, and “modification-dependent protein catabolic process” (Figure S5F). Furthermore, we generated Sankey diagrams to analyze several biological processes and cellular components closely related to the ER. This analysis revealed several differential glycoproteins that could serve as candidate molecules for RPN1 depletion leading to ERS (Figure 5J). Thus, based on the above results, we hypothesized that RPN1 depletion leads to decreased glycosylation of ER chaperone proteins, which in turn causes ERS.

### RPN1 deletion triggers ERS and causes ER dysfunction

**Figure 6:**
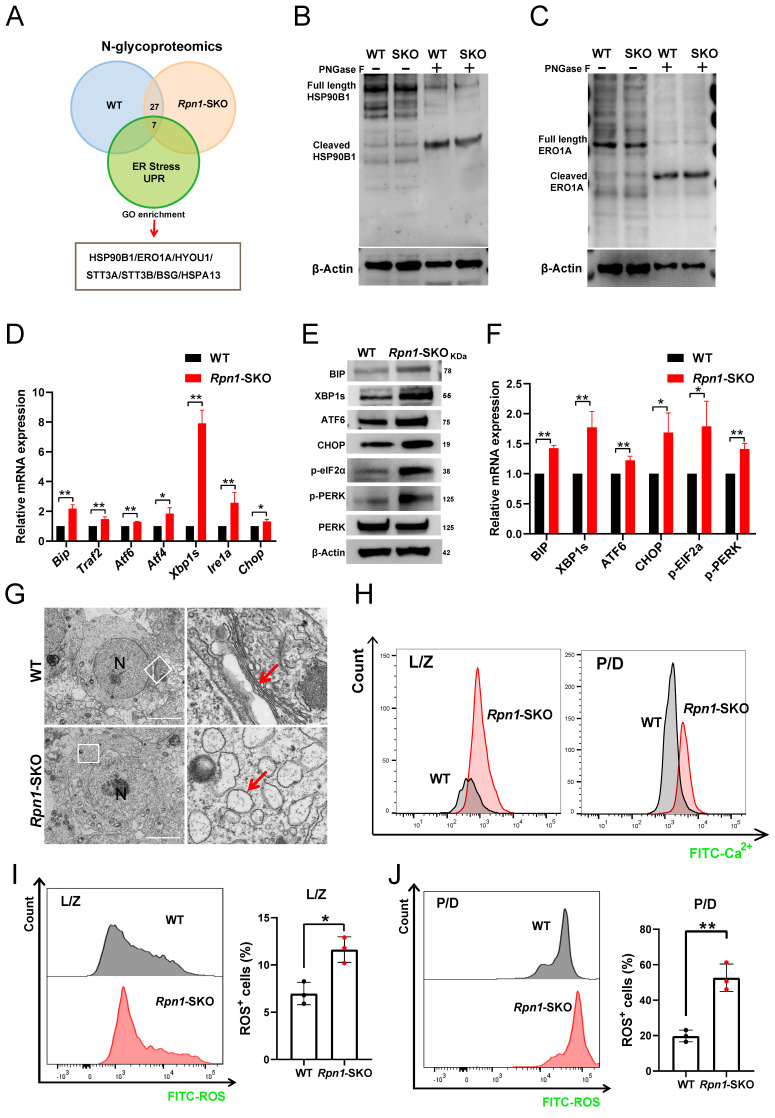
** RPN1 deficiency induces ERS of spermatocytes.** A. Venn diagram showed the number of proteins identified by N-glycoproteomics from WT or *Rpn1*-SKO, as well as the proteins belonging to GO enrichment terms of ERS and UPR. **B-C.** WB detection of key protein glycosylation levels in adult WT and *Rpn1*-SKO testes. **D.** Analysis of the expression level of ERS related genes by RT-qPCR. *β-Actin* served as loading control. Data represent means ± SEM. *p < 0.05, **p < 0.01 by Student's t-test. **E-F.** WB analysis of ERS related protein levels and quantification. β-Actin served as loading control. Data represent means ± SEM, *p < 0.05, **p < 0.01 by Student's t-test. **G.** TEM imaging of ER architecture in testicular sections from 2M WT and *Rpn1*-SKO mice. N, nucleus. Scale bar, 5 µm. **H.** Quantification of Fluo-4 AM fluorescence in L/Z and P/D spermatocytes of WT and *Rpn1*-SKO mice measured by FACS. **I-J.** Quantification of CM-H2DCFDA fluorescence in L/Z and P/D spermatocytes of WT and *Rpn1*-SKO was measured by FACS. Data represent means ± SEM, *p < 0.05, **p < 0.01 by Student's t-test.

To identify the glycoproteins involved in RPN1 regulation of ERS, we focused on glycoproteins with decreased glycosylation in our N-glycoproteomics dataset and found seven that were related to the GO terms, UPR and ERS^27^, ^51^-^55^ (Figure 6A). To further verify the glycosylation levels of these proteins identified in the N-glycoproteome, we conducted WB analyses of several ERS-related glycoproteins in WT and *Rpn1*-SKO testes, treated with or without the recombinant glycosidase, peptide-N-glycosidase F (PNGase F), which can remove N-linked oligosaccharides from polypeptides^56^. We observed the shift to lower molecular weights of these proteins following exposure to PNGase F, indicating the heavily glycosylated status of them in testes. However, no changes in the molecular weight of these proteins were observed in the *Rpn1*-SKO testes. We propose that RPN1 primarily mediates specific glycosylation modifications on these proteins rather than all glycosylation sites. Moreover, WB analysis provides only a rough estimate of the overall glycosylation level and may not accurately reflect the glycosylation status at specific sites (Figure 6B and 6C and Figure S6A).

We then further investigated the effects of RPN1 deletion on ERS in mouse testes. BIP is a well-known marker of ERS that plays a crucial role in activation of the UPR. Under ERS conditions, BIP dissociates from receptor proteins in the ER, such as IRE1, PERK, and ATF6, resulting in their activation and subsequent initiation of the UPR. Since BIP is a key upstream molecule in three major branches of the UPR pathway, its upregulation is commonly used as a marker of ERS^57^. The results of RT-qPCR, WB, and IF showed that BIP was indeed significantly upregulated in *Rpn1*-SKO testes (Figures 6D-6F and Figure S6B), suggesting severe ERS. To further determine which specific ERS signaling pathway was activated, we examined mRNA and protein levels of key molecules associated with each branch of the ERS pathway. We found that *Atf6* (in the ATF6 branch), *Atf4* and *Chop* (in the PERK branch), as well as *Traf2* and *Xbp1s* (in the IRE1 branch) were all significantly upregulated in *Rpn1*-SKO testes (Figure 6D). The induction of ERS was further confirmed by WB analysis of these markers (Figure 6E and 6F). Given our above findings that RPN1 deletion resulted in ERS in mouse testes, we sought to directly observe possible defects in the ER of spermatocytes with RPN1 deletion. TEM imaging of ER morphology and architecture in spermatocytes revealed that the morphology of the ER in WT mouse spermatocytes typically remains stable during different stages of development, exhibiting a relatively uniform network of membranous structures. However, in *Rpn1*-SKO mouse spermatocytes at various developmental stages, the ER membranes become swollen or distorted, displaying vesicular structures, and even rupturing (Red arrow, Figure 6G and Figure S6C). As ERS can disrupt calcium homeostasis, thereby negatively impacting protein folding and transport^26^, we quantified intracellular Ca^2+^ levels in spermatocytes. FACS analysis with the fluorescent Ca^2+^ indicator, Rhod-4 AM showed that intracellular Ca^2+^ levels were indeed significantly increased in L/Z and P/D spermatocytes of *Rpn1*-SKO mice, suggesting disrupted ER function (Figure 6H). As ERS can lead to ROS generation and apoptosis^30^, we also measured ROS in spermatocytes by FACS and found significantly increased ROS levels in *Rpn1*-SKO spermatocytes compared to that in WT (Figure 6I and 6J). Subsequent detection of several apoptotic signaling proteins by WB showed that the pro-apoptotic proteins, BAX, Caspase3, and cleaved Caspase3, were all significantly elevated, while the anti-apoptotic protein, BCL2, was relatively decreased in *Rpn1*-SKO testes compared with controls (Figure S6D). These findings indicate that RPN1 deficiency leads to reduced glycosylation levels of ER-associated glycoproteins, causing ERS and increased apoptosis in spermatocytes. While ERS is a known source of ROS, we cannot exclude the possibility that ROS elevation observed in *Rpn1*-SKO spermatocytes may also be influenced by meiotic defects and subsequent apoptosis. Collectively, these observations may suggest that both ERS and meiotic failure contribute to the cellular stress responses that ultimately lead to spermatocyte apoptosis and male infertility.

## Discussion

In the male reproductive system, several crucial proteins are known to undergo N-glycosylation modification. However, the specific role and mechanism of these modifications in mammalian spermatogenesis and the overall male reproductive system remain unclear. Here, we found that knockout of the N-glycosylation-associated protein, RPN1, results in abnormal spermatocyte meiosis and complete infertility in male mice. N-glycoproteomic analysis revealed that the deletion of RPN1 leads to a significant decrease in glycosylation levels of ER-associated proteins, subsequently affecting the structure and function of ER. Our findings provide new insights into the relationship between N-glycosylation modification and meiosis. Further investigation of this relationship can facilitate advances in developing treatments for male infertility.

Spermatogenesis involves complex PTMs, such as phosphorylation, acetylation, glycosylation, and others, which play crucial regulatory roles in spermatogenesis^58^-^60^. In recent years, advances in mass spectrometry, glycomics and glycoproteomics, have shown that N-glycosylation modifications play an essential role in spermatogenesis in mice^10^, ^61^, ^62^. The impairments of the glycosylation process can disrupt the activity of glycoproteins and other glycosylated molecules, potentially resulting in CDGs^9^. In the male reproductive system, many functional proteins are known to undergo glycosylation modification. For example, in mice with knockout for the multifunctional blood-testis barrier glycoprotein, *Basigin* (BSG), CDH2 (N-Cadherin), a calcium-dependent cell adhesion molecule, was also expressed as significantly lower levels, suggesting that reduced BSG and CDH2 levels could potentially interfere with interactions between germ cells and Sertoli cells, leading to arrest of spermatogenesis^62^. Other research has shown that spermatocyte-specific *Mgat1* knockout in *Stra8-GFPCre* mice prevents sperm formation, resulting in male infertility^10^. Most glycosylation disorders, or N-linked glycosylation disorders, are associated with abnormal synthesis of N-linked oligosaccharides that bind to Asn residues on proteins^9^. As an auxiliary subunit of the OST complex, RPN1 plays an important role in enhancing N-glycosylation modifications. However, the function of RPN1 in the male reproductive system has not been reported. In our study, we found that RPN1 deficiency results in abnormal spermatocytes in meiosis and complete male sterility. These results suggested that RPN1 depletion might also cause CDGs, and therefore RPN1 might serve as reliable biomarker for some diseases.

The ER is capable of dynamically adjusting to increased protein-folding demands. Nevertheless, external stimuli and changes in intracellular homeostasis can result in protein misfolding within the ER^63^. In addition, protein folding activity in the ER can also regulate downstream adaptive or apoptotic responses. Abnormalities in OST subunits can result in protein hypoglycosylation, leading to protein misfolding. The accumulation of these misfolded proteins can disrupt ER homeostasis, eventually resulting in a disproportionately high protein folding load relative to ER folding capacity^47^, ^64^. Although RPN1 has been shown to promote N-glycosylation, its corresponding mechanism and role in spermatogenesis remain unclear. N-glycoproteomics analysis in the current study revealed that *Rpn1* knockout leads to significantly reduced glycosylation levels of seven proteins associated with ER chaperones, including HYOU1, ERO1A and HSP90B1. Previous studies shown that deficiency for fucosyltransferase 2 leads to reduced HYOU1 glycosylation, which can trigger ERS^53^. As important molecular chaperones in the ER, depletion of HSP90B1 or ERO1A can both lead to ERS, but their relationship with N-glycosylation remains unknown^65^, ^66^. Our findings indicated that the ERS marker, BIP, and other key molecules in the three UPR pathways were all significantly activated in *Rpn1-*SKO mouse testes. These results suggest that impaired glycosylation of ER molecular chaperone proteins can induce ERS, which could mediate the *Rpn1* deficiency phenotype, thus highlighting the crucial role of *Rpn1* in correct protein folding and synthesis during spermatocyte development.

Early studies on RPN1 suggested that it primarily functions in the N-glycosylation of certain single-pass transmembrane proteins, while having little to no impact on the glycosylation of multi-pass transmembrane or secretory proteins^67^. However, our N-glycoproteomics analysis revealed glycosylation alterations in three luminal ER proteins: HSP90B1, HYOU1, and ERO1A, which drew our significant interest. To investigate the glycosylation site alterations of these proteins, we analyzed their secondary spectra and identified distinct glycosylation sites in all three proteins. Importantly, the altered sites fully conformed to the N-X-T/S consensus sequence (Table S4). To validate the results of our N-glycosylation proteomics analysis, we repeated the N-glycosylation sequencing using another core facility (JingJie Biotechnology Co., Ltd., Hangzhou, Accession number: PXD051379), and found that glycosylation sites on HSP90B1 and HYOU1 indeed changed significantly, while the differences in ERO1A glycosylation did not reach significance, but did undergo changes at its N-glycosylation sites. In addition, we propose that previous studies, which relied primarily on WB to validate glycosylation changes in a limited set of single-pass, multi-pass, and secretory proteins, may have overlooked certain glycosylation targets due to the lack of a comprehensive and systematic approach^67^. By employing a global N-glycoproteomics strategy coupled with tandem mass spectrometry in this study, we were able to identify glycosylation site changes with greater sensitivity and reliability.

In addition to our N-glycosylation analysis, we conducted proteomic profiling, which identified several downregulated proteins that were enriched in the ER, providing further evidence of ER dysfunction in Rpn1-deficient spermatocytes (Figure S5A and S5B). We also found that several ER chaperone proteins are upregulated in *Rpn1*-SKO testes, suggesting the occurrence of ERS. These findings indicate that RPN1 deficiency disrupts protein quality control pathways in the ER, which in turn may affect protein folding processes. Although our results show the induction of ERS, we did not directly assess the impacts of *Rpn1* deficiency on protein folding. Given RPN1 is known to contribute to glycoprotein quality control, future studies should investigate the possible role of RPN1 in protein folding through protein misfolding assays, monitoring ER chaperone dynamics, and measuring protein folding efficiency in Rpn1-deficient cells. Such studies could expand our current understanding of the role of RPN1 in maintaining protein homeostasis and ER function.

Although some round spermatids are present in *Rpn1*-SKO testes, it was crucial to investigate the critical stages of meiotic division in these testes more thoroughly. Examination of SYCP3 and SYCP1 expression patterns in our spermatocyte spreads showed that significantly fewer *Rpn1*-SKO spermatocytes were in the pachytene and diplotene stages compared to those of WT mice, but instead accumulated in the zygotene stage. Based on the expression patterns of markers such as SYCP3, rH2AX, RPA2, and DMC1, we found that the *Rpn1*-deficient testes contained a large number of pachytene spermatocytes with abnormalities in synapsis and homologous recombination. This finding is consistent with a previous study showing that pachytene spermatocytes in germ cells of protein disulfide isomerase (PDI) knockout mice, which catalyze the formation of disulfide bonds, also exhibit numerous synaptic and recombination abnormalities^68^. We were also interested in possible explanations as to why we detected only a small number of diplotene cells and round spermatids in *Rpn1*-SKO mice. It is possible that although ER structure and function are impaired in *Rpn1*-SKO spermatocytes, the cells might correct misfolded proteins through additional mechanisms to complete meiosis. Additionally, MLH1, a marker protein for crossover, was absent in *Rpn1*-SKO spermatocytes, indicating that crossovers occur infrequently during the mid-pachytene stage. It is known that crossover will not happen if the chromosomes are in a condition of asynapsis^5^. We further demonstrated that the meiotic obstruction ultimately resulted in the apoptosis of the *Rpn1*-SKO spermatocytes, which explained the testicular atrophy observed in the mutant mice. Based on these data, we conclude that RPN1 may impair protein folding by directly affecting the morphology and function of the ER, thereby indirectly impacting meiosis.

In summary, our study provides a comprehensive analysis of the function of RPN1 in spermatogenesis in mice. We provide convincing evidence that *Rpn1* deficiency severely affected ER functions in spermatocytes, which further disrupted meiotic events and progression, and eventually failure of spermatogenesis. Our current research will establish a solid foundation for future investigations on the roles of various modifications in spermatogenesis.

## Supplementary Material

Supplementary figures and tables, data.

## Abbreviations

RPN1: ribophorin 1
ERS: endoplasmic reticulum stress
DSBs: DNA double strand breaks
CDGs: congenital disorders of glycosylation
OST: oligosaccharyltransferase
UPR: unfolded protein response pathway
PTM: post-translational modifications
PD14: postnatal day 14
Con A: concanavalin A
WB: western blotting
RT-qPCR: reverse-transcription quantitative real-time PCR
TEM: transmission electron microscopy
PNA: peanut agglutinin
PNGase F: Peptide-N-glycosidase F
ROS: reactive oxygen species
