# Supplementary material for: Dysregulation of N-glycosylation by Rpn1 knockout in spermatocytes induces male infertility via endoplasmic reticulum stress in mice
Source: Int J Biol Sci. 2025 Mar 3;21(5):2360–79. doi: 10.7150/ijbs.106468 (PMC11900820; doi:10.7150/ijbs.106468)
Supplement: Supplementary file 1 — Supplementary figures and tables, data. [file ijbsv21p2360s1.pdf]

**Table S1. PCR primer sequence**

| Genes | Primer sequence |
|---|---|
| *Stra8-GFPcre* | F:ACTCCAAGCACTGGGCAGAA |
| | R1:GCCACCATAGCAGCATCAAA |
| | R2:CGTTTACGTCGCCGTCCAG |
| *Vasa-Cre* | F:AAGAACCTGATGGACATGTTCAG |
| | R:CTGATCCTGGCAATTTCGG |
| *Rpn1* | R:GGCAGGGAGATATATCAGTGGGTAAA |
| | F1:GGGTTCTCTGTGAAATTGTTCATGTTC |
| | F2:GGACCATTTAGGAAGTGAATGTGGA |

**PCR Reaction Component**

| Reaction component | Volume (μl) |
|---|---|
| 2 × Taq Master Mix, Dye Plus, (Vazyme P112-03) | 12.5 |
| ddH2O | 9.5 |
| Primer F(10pmol/μl) | 1 |
| Primer R(10pmol/μl) | 1 |
| Template | 1 |

**PCR program**

| Temp. | Time | Cycle |
|---|---|---|
| 95℃ | 5min | |
| 98℃ | 30s | 20× |
| 65℃（-0.5℃/cycle） | 30s | |
| 72℃ | 45s | |
| 98℃ | 30s | 20× |
| 55℃ | 30s | |
| 72℃ | 45s | |

| | | |
|---|---|---|
| 72℃ | 5min | |
| 10℃ | hold | |

**RT-qPCR primer sequence**

| | |
|---|---|
| *Rpn1* | F:GCTCCACATCACGAGCCAG |
| | R:CAGTTTCCACAACGACCGAGA |
| *Xbp1s* | F:TGCTGAGTCCGCAGCAGGTG |
| | R:GCTGGCAGGCTCTGGGGAAG |
| *Perk* | F:TGGTGTCATCCAGCCTTAGCAA |
| | R:CATGCTTTCACGGTCTTGGTCC |
| *Ire1α* | F:ACTTTGTCATCGGCCTTTGCAG |
| | R:AGTGAGGCCGCATAGTCAAAGT |
| *Bip* | F:TCTGTGCAGCAGGACATCAAGT |
| | R:GTCTTTGTTTGCCCACCTCCAA |
| *Chop* | F:GGGAAACAGCGCATGAAGGA |
| | R:GCGTGATGGTGCTGGGTACA |
| *Atf6* | F:TGGGCAGGACTATGAAGTAATG |
| | R:CAACGACTCAGGGATGGTGCTG |
| *Atf4* | F:ATGGCGCTCTTCACGAAATC |
| | R:ACTGGTCGAAGGGGTCATCAA |
| *Traf2* | F:AGAGAGTAGTTCGGCCTTTCC |
| | R:GTGCATCCATCATTGGGACAG |
| *β-Actin* | F:GGGTCAGAAGGATTCCTATG |
| | R:GGTCTCAAACATGATCTGGG |
| *Gapdh* | F:GTCATTGAGAGCAATGCCAG |
| | R:GTGTTGCTACCCCCAATGTG |

**Table S2. Antibody information**

| Primary antibodies | Vendor | Dilution | Source |
|---|---|---|---|
| RPN1 (IF/WB) | Santa Cruz Biotechnology (SC48367) | 1:200/1:1000 | Mouse |
| γH2AX(Ser139) (IF/WB) | Millipore (05-636) | 1:1000 | Mouse |
| β-Actin (WB) | Proteintech(66009-1-Ig) | 1:1000 | Rabbit |
| MVH (IF/WB) | Abcam (ab13840) | 1:1000 | Rabbit |
| DMC1 (IF) | Santa Cruz Biotechnology (sc-22768) | 1:500 | Rabbit |
| RAD51 (IF) | Thermo Fisher Scientific (PA5-27195) | 1:500 | Rabbit |
| SYCP3 (IF) | Abcam (ab97672) | 1:500 | Mouse |
| SYCP3 (IF) | Abcam (ab15093) | 1:500 | Rabbit |
| SYCP1 (IF) | Abcam (ab15090) | 1:500 | Rabbit |
| LAMIN B1 (WB) | Proteintech(12987-1-AP) | 1:1000 | Rabbit |
| PLZF (IF/WB) | Santa Cruz Biotechnology (SC28319) | 1:500 | Mouse |
| c-Kit (IF) | Proteintech (18696-1-AP) | 1:500 | Rabbit |
| SOX9 (IF/WB) | Millipore (3389351) | 1:500 | Rabbit |
| STRA8 (IF/WB) | Abcam (ab49602) | 1:500 | Mouse |
| H1t (IF) | Abcam (ab81498) | 1:500 | Rabbit |
| MLH1 (IF) | Abcam (ab92312) | 1:500 | Rabbit |
| RPA2 (IF) | Abcam (ab76420) | 1:500 | Rabbit |
| BIP (WB) | Proteintech (11587-1-AP) | 1:1000 | Rabbit |
| XBP1s (WB) | Proteintech (24868-1-AP) | 1:1000 | Rabbit |
| ATF6 (WB) | Proteintech (24169-1-AP) | 1:1000 | Rabbit |
| CHOP (WB) | Proteintech (15204-1-AP) | 1:1000 | Rabbit |
| p-eIF2α (WB) | Cell Signaling Technology | 1:1000 | Rabbit |

| | | | |
|---|---|---|---|
| | (D9G8) | | |
| p-PERK (WB) | Affinity (DF7576) | 1:1000 | Rabbit |
| PERK (WB) | Abmart (MA8131S) | 1:1000 | Rabbit |
| HSP90B1 (WB) | Santa Cruz Biotechnology (sc-517405) | 1:1000 | Mouse |
| HYOU1 (WB) | Santa Cruz Biotechnology (sc-398224) | 1:1000 | Rabbit |
| ERO1A (WB) | Proteintech (12007-1-AP) | 1:1000 | Rabbit |
| BSG (WB) | Proteintech (11989-1-AP) | 1:1000 | Rabbit |
| STT3A (WB) | Proteintech (12034-1-AP) | 1:1000 | Rabbit |
| STT3B (WB) | Proteintech (15323-1-AP) | 1:1000 | Rabbit |
| DAD1 (WB) | Abclonal (A14723) | 1:1000 | Rabbit |
| DDOST (WB) | Abclonal (A9056) | 1:1000 | Rabbit |
| BAX (WB) | Proteintech (50599-2-Ig) | 1:1000 | Rabbit |
| BCL2 (WB) | Proteintech (26593-1-AP) | 1:1000 | Rabbit |
| Caspase 3 (WB) | Cell Signaling Technology (9662) | 1:1000 | Rabbit |
| Cleaved Caspase 3 (WB) | Cell Signaling Technology (9664) | 1:1000 | Rabbit |
| Alexa Fluor 488 Goat anti-Mouse IgG | Abcam (ab150117) | IF (1:500) | Goat |
| Alexa Fluor 594 Goat anti-Rabbit IgG | Abcam (ab150080) | IF (1:500) | Goat |
| Alexa Fluor 594 Goat anti-Mouse IgG | Abcam (ab150120) | IF (1:500) | Goat |
| Alexa Fluor 488 Goat anti-Rabbit IgG | Abcam (ab150077) | IF (1:500) | Goat |
| HRP-conjugated goat anti- Mouse IgG | Beyotime (A0216) | WB (1:1000) | Goat |

| | | | |
|---|---|---|---|
| HRP-conjugated goat anti- rabbit IgG (WB) | Beyotime (A0258) | WB (1:1000) | Goat |

**Table S3. Alterations in Protein Glycosylation Site**

| Protein name | Glycosylation Sites | Observed Modification | Assigned Modification | SKO/WT Ratio | Regulated Type |
|---|---|---|---|---|---|
| ITGB5 | N[347] | HexNAc(2)Hex(8) | 1702.5813 | 0.615999028 | Down |
| HSP90B1 | N[62] | HexNAc(2)Hex(6) | 1378.4757 | 0.372636373 | Down |
| HSP90B1 | N[217] | HexNAc(2)Hex(8) | 1702.5813 | 0.650323658 | Down |
| ITGB1 | N[669] | HexNAc(2)Hex(6) | 1378.4757 | 0.635035799 | Down |
| SLC3A2 | N[385] | HexNAc(2)Hex(7) | 1540.5285 | 0.660697221 | Down |
| SLC3A2 | N[385] | HexNAc(2)Hex(9) | 1864.6341 | 0.623229373 | Down |
| LAMP2 | N[322] | HexNAc(2)Hex(6) | 1378.4757 | 0.507568378 | Down |
| CTSD | N[134] | HexNAc(2)Hex(7) | 1540.5285 | 0.256563492 | Down |
| BSG | N[160] | HexNAc(2)Hex(9) | 1864.6341 | 0.476613385 | Down |
| LAMA1 | N[45] | HexNAc(2)Hex(9) | 1864.6341 | 0.262010587 | Down |
| ABCA1 | N[1637] | HexNAc(2)Hex(9) | 1864.6341 | 0.12599944 | Down |
| STT3A | N[537] | HexNAc(2)Hex(9) | 1864.6341 | 0.23900947 | Down |
| LAMA4 | N[308] | HexNAc(4)Hex(5)Fuc(1) | 1768.6395 | 0.570055025 | Down |
| COl6A2 | N[912] | HexNAc(4)Hex(5)Fuc(1) | 1768.6395 | 0.127530896 | Down |
| HSPG2 | N[3098] | HexNAc(3)Hex(5)Fuc(1) | 1565.5601 | 0.129190029 | Down |
| STT3B | N[613] | HexNAc(2)Hex(9) | 1864.6341 | 0.240063169 | Down |
| HSPA13 | N[184] | HexNAc(2)Hex(8) | 1702.5813 | 0.361818703 | Down |
| COLGALlT1 | N[376] | HexNAc(4)Hex(4) | 1460.5288 | 0.585103856 | Down |
| ERO1A | N[276] | HexNAc(2)Hex(8) | 1702.5813 | 0.550938518 | Down |
| TMED4 | N[117] | HexNAc(5)Hex(3)Fuc(1) | 1647.6132 | 0.38151888 | Down |
| MCAM | N[58] | HexNAc(5)Hex(4)Fuc(1) NeuAc(1) | 2100.7615 | 0.528611452 | Down |
| LRP1 | N[1512] | HexNAc(2)Hex(6) | 1378.4757 | 0.614586827 | Down |

| | | | | | |
|---|---|---|---|---|---|
| TMED9 | N[125] | HexNAc(4)Hex(3)Fuc(1) | 1444.5338 | 0.66556221 | Down |
| CRTAP | N[362] | HexNAc(2)Hex(8) | 1702.5813 | 0.340694912 | Down |
| DPEP3 | N[240] | HexNAc(4)Hex(4)Fuc(1) | 1606.5867 | 0.251143261 | Down |
| HYOU1 | N[515] | HexNAc(2)Hex(9) | 1864.6341 | 0.656782783 | Down |
| HYOU1 | N[830] | HexNAc(2)Hex(7) | 1540.5285 | 0.502468366 | Down |
| HYOU1 | N[830] | HexNAc(2)Hex(8) | 1702.5813 | 0.485332445 | Down |
| TEX101 | N[45] | HexNAc(4)Hex(4)Fuc(1) | 1606.5867 | 0.301223764 | Down |
| TEX101 | N[45] | HexNAc(4)Hex(5)Fuc(1) | 1768.6395 | 0.142273473 | Down |
| TEX101 | N[134] | HexNAc(2)Hex(7) | 1540.5285 | 0.346869026 | Down |
| TEX101 | N[134] | HexNAc(2)Hex(8) | 1702.5813 | 0.361086511 | Down |
| TEX101 | N[160] | HexNAc(2)Hex(8) | 1702.5813 | 0.484853608 | Down |
| NUP210 | N[405] | HexNAc(2)Hex(9) | 1864.6341 | 0.482313481 | Down |
| PLOD2 | N[696] | HexNAc(2)Hex(7) | 1540.5285 | 0.494405884 | Down |

14

| Protein name | Glycosylation Sites | Observed Modification | Assigned Modification | SKO/WT Ratio | Regulated Type |
|---|---|---|---|---|---|
| HSP90B1 | N[445] | HexNAc(2)Hex(8) | 1702.5813 | 3.792752041 | Up |
| HSP90B1 | N[481] | HexNAc(2)Hex(8) | 1702.5813 | 2.282822295 | Up |
| HSP90B1 | N[481] | HexNAc(2)Hex(9) | 1864.6341 | 2.999030985 | Up |
| HSP90B1 | N[502] | HexNAc(2)Hex(7) | 1540.5285 | 3.904399469 | Up |
| ACP2 | N[267] | HexNAc(2)Hex(9) | 1864.6341 | 2.075285066 | Up |
| BGN | N[312] | HexNAc(5)Hex(5)Fuc(1) | 1971.7189 | 1.704358073 | Up |
| LUM | N[127] | HexNAc(4)Hex(6)NeuAc(1) | 2075.7298 | 2.042297464 | Up |
| GXYlT1 | N[201] | HexNAc(2)Hex(9) | 1864.6341 | 1.622291229 | Up |
| ERLIN2 | N[106] | HexNAc(2)Hex(9) | 1864.6341 | 11.24429231 | Up |
| BTD | N[326] | HexNAc(2)Hex(6) | 1378.4757 | 7.710842273 | Up |
| LRP1 | N[1826] | HexNAc(2)Hex(7) | 1540.5285 | 1.535336334 | Up |
| LRP1 | N[3840] | HexNAc(2)Hex(7) | 1540.5285 | 1.888928898 | Up |
| TEX101 | N[45] | HexNAc(5)Hex(4)Fuc(1) | 1809.666 | 1.983835733 | Up |

| | | | | | |
|---|---|---|---|---|---|
| TEX101 | N[160] | HexNAc(2)Hex(10) | 2026.6869 | 2.046098645 | Up |
| SEMA7A | N[256] | HexNAc(2)Hex(8) | 1702.5813 | 16.49727389 | Up |

**Table S4. The secondary spectra of proteins.**

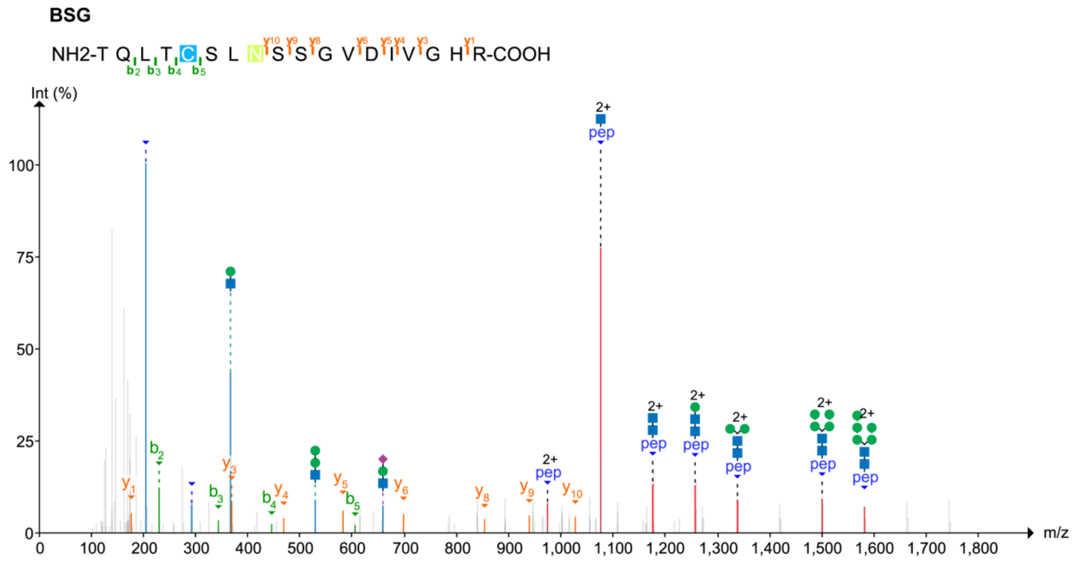


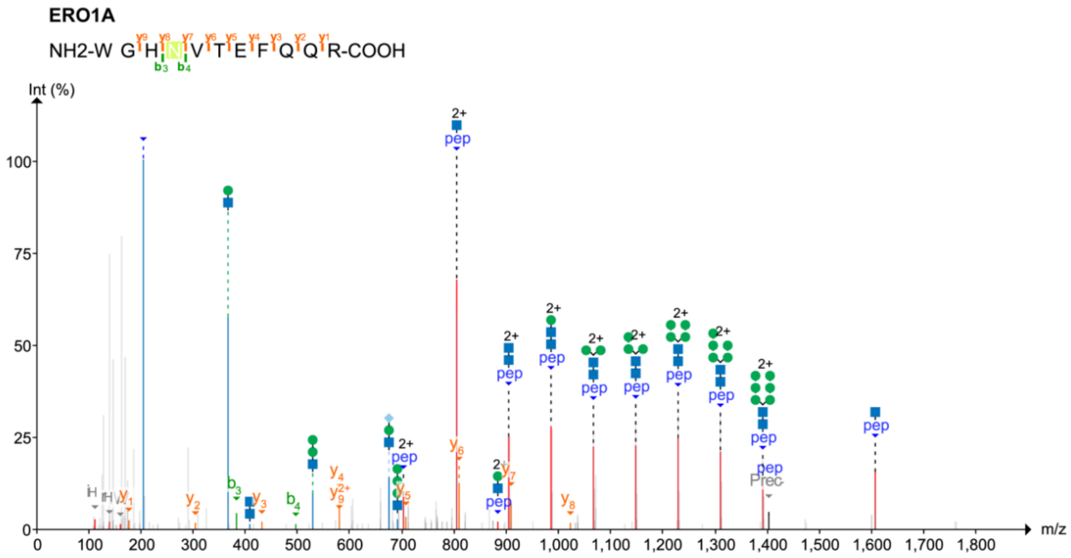

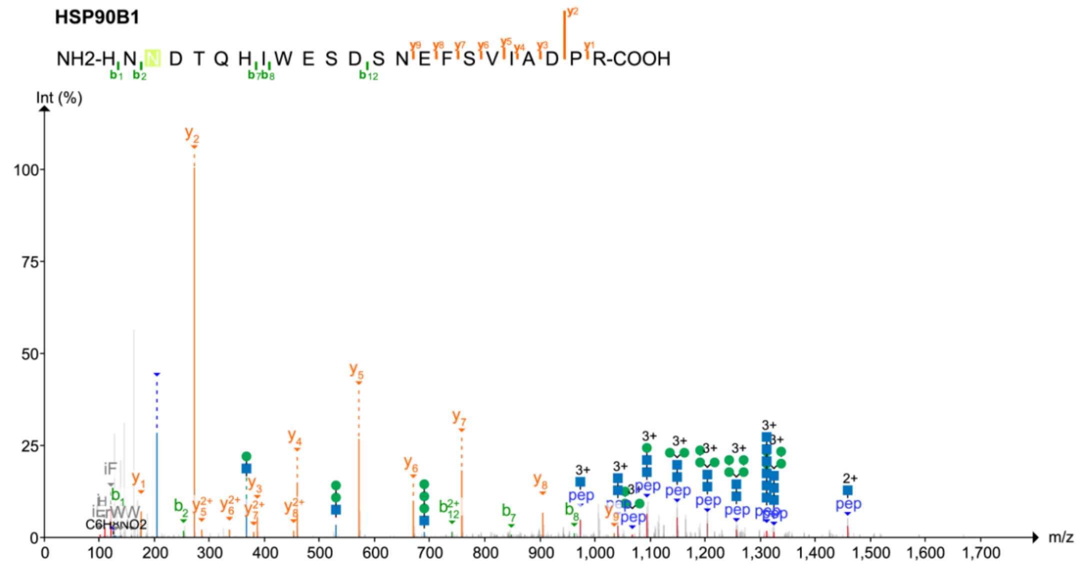
HSP90B1
NH2-HNNDTQHIWESDSNEFSVIADPR-COOH
Int (%)
m/z

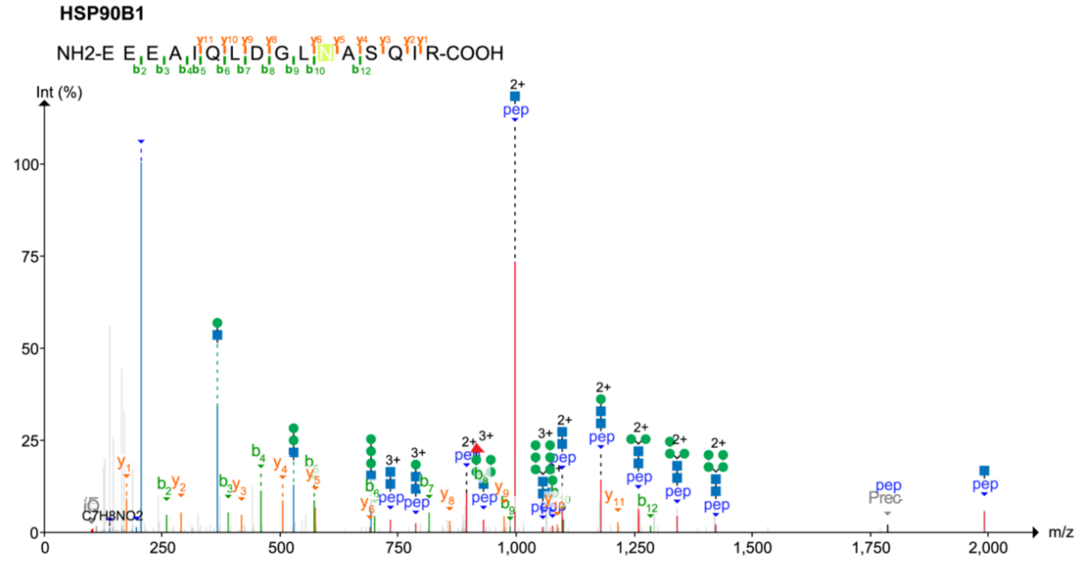
HSP90B1
NH2-EEAIQLDGLNASQIR-COOH
Int (%)
m/z

**HSPA13**

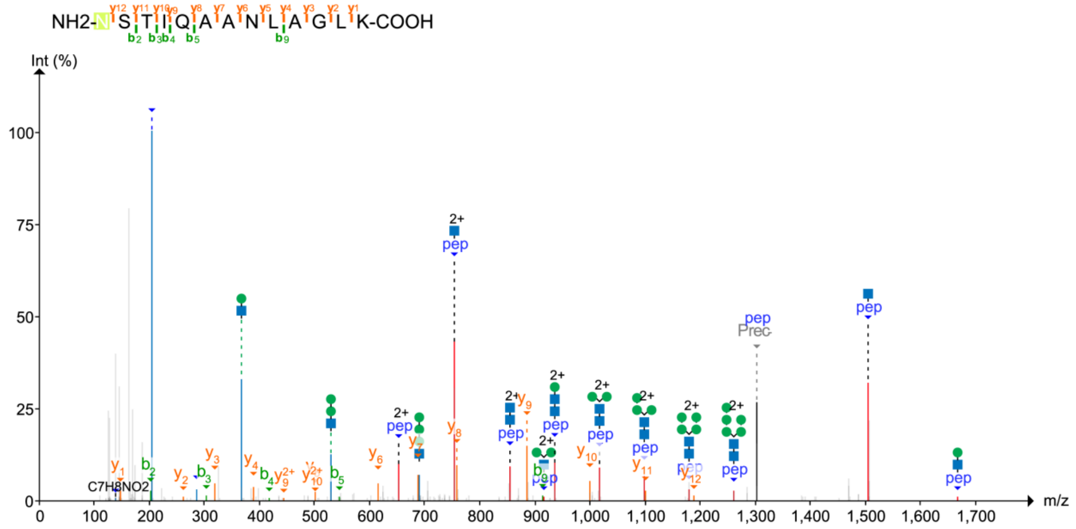


**HYOU1**

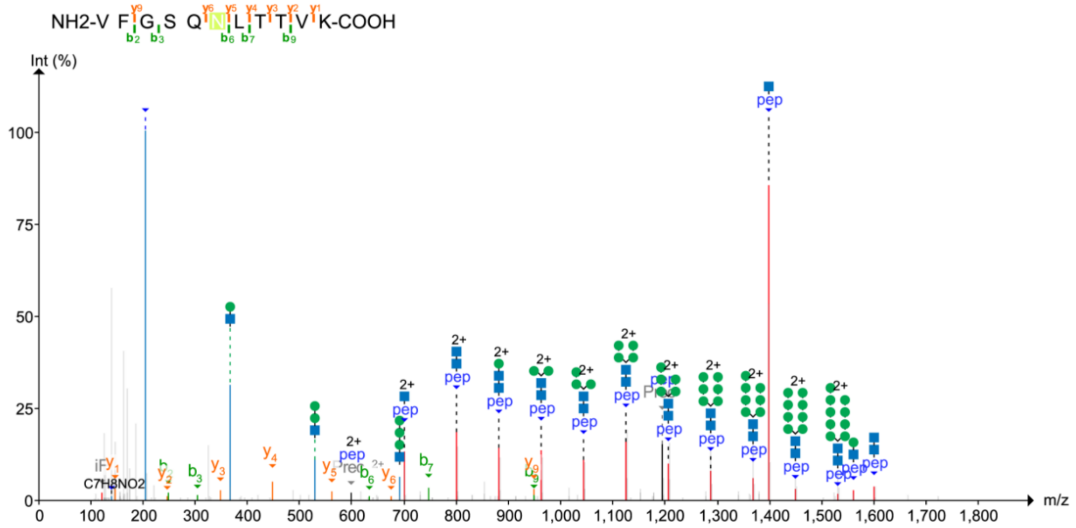

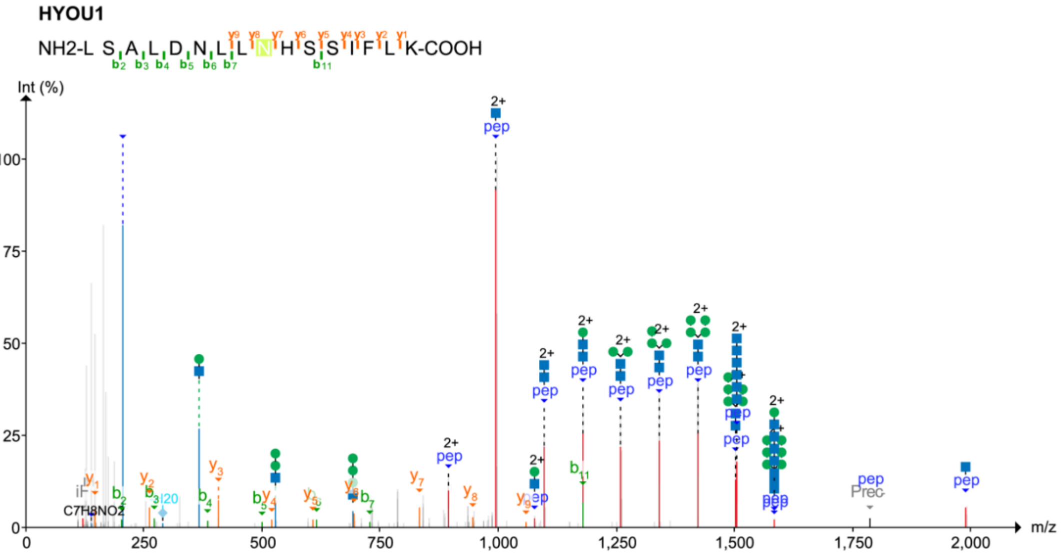
HYOU1
NH2-L S A L D N L L N H S S I F L K-COOH
Int (%)
m/z

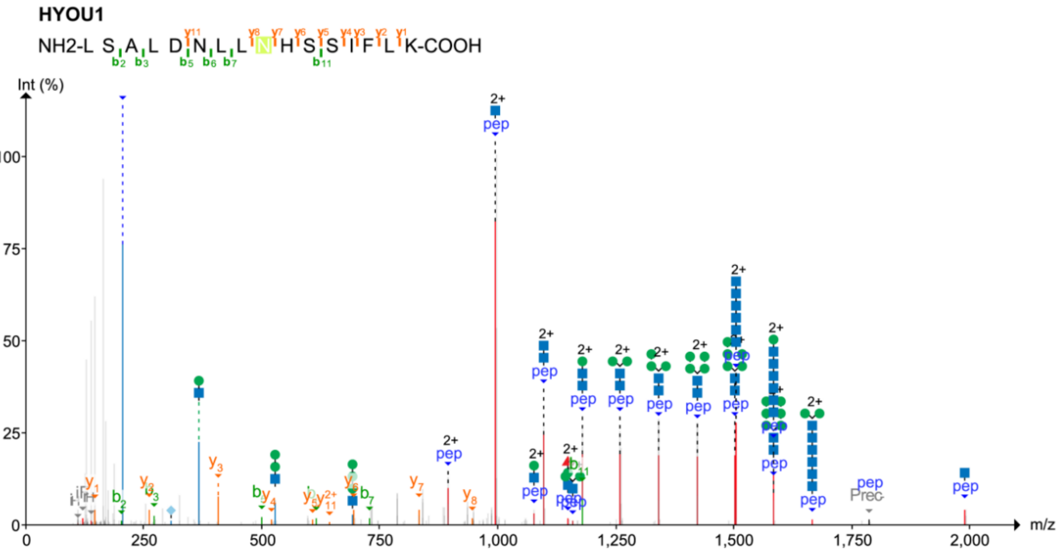
HYOU1
NH2-L S A L D N L L N H S S I F L K-COOH
Int (%)
m/z

**STT3A**

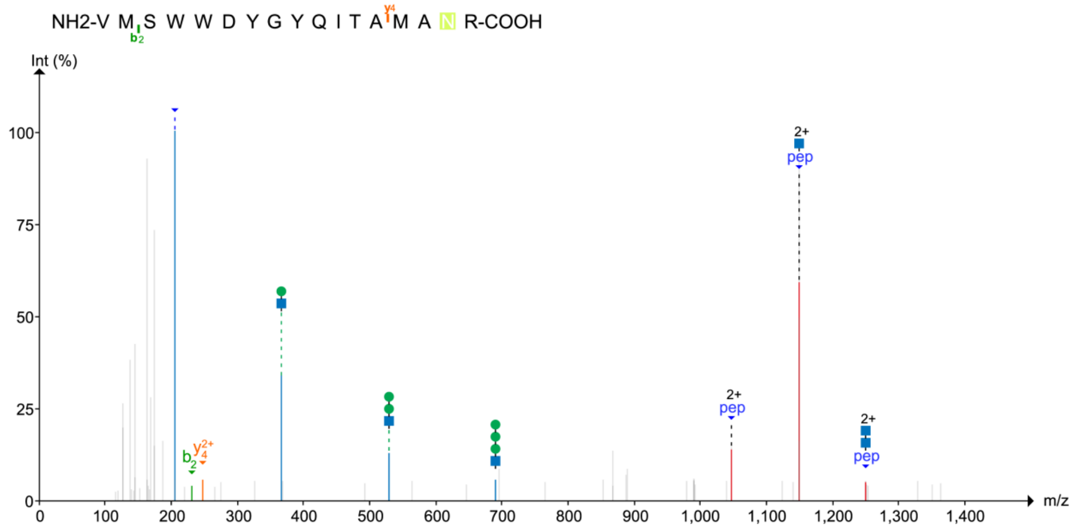
NH2-V M S W W D Y G Y Q I T A M A N R-COOH
Int (%)
m/z
pep
2+

**STT3B**

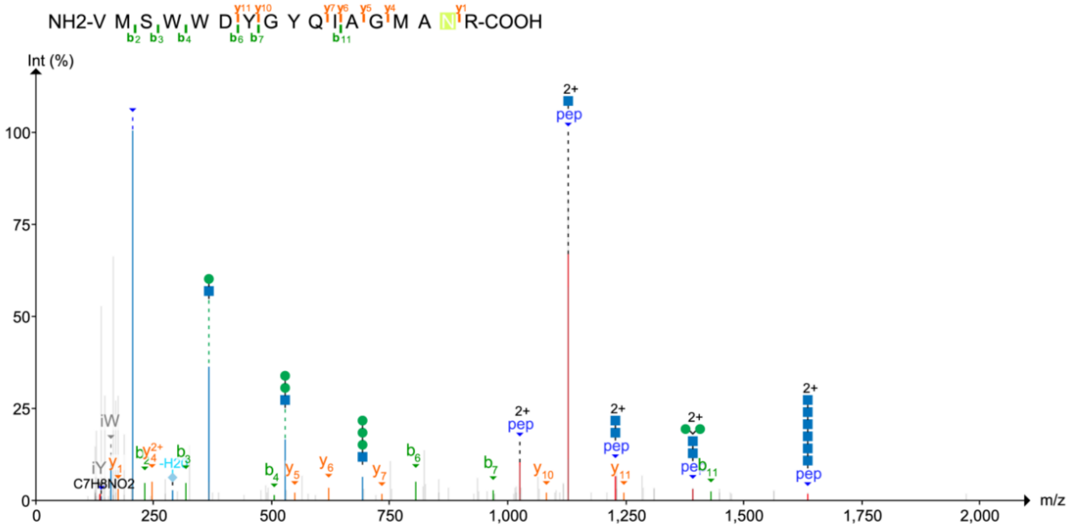
NH2-V M S W W D Y G Y Q I A G M A N R-COOH
Int (%)
m/z
pep
2+
C7H8NO2
-H2O

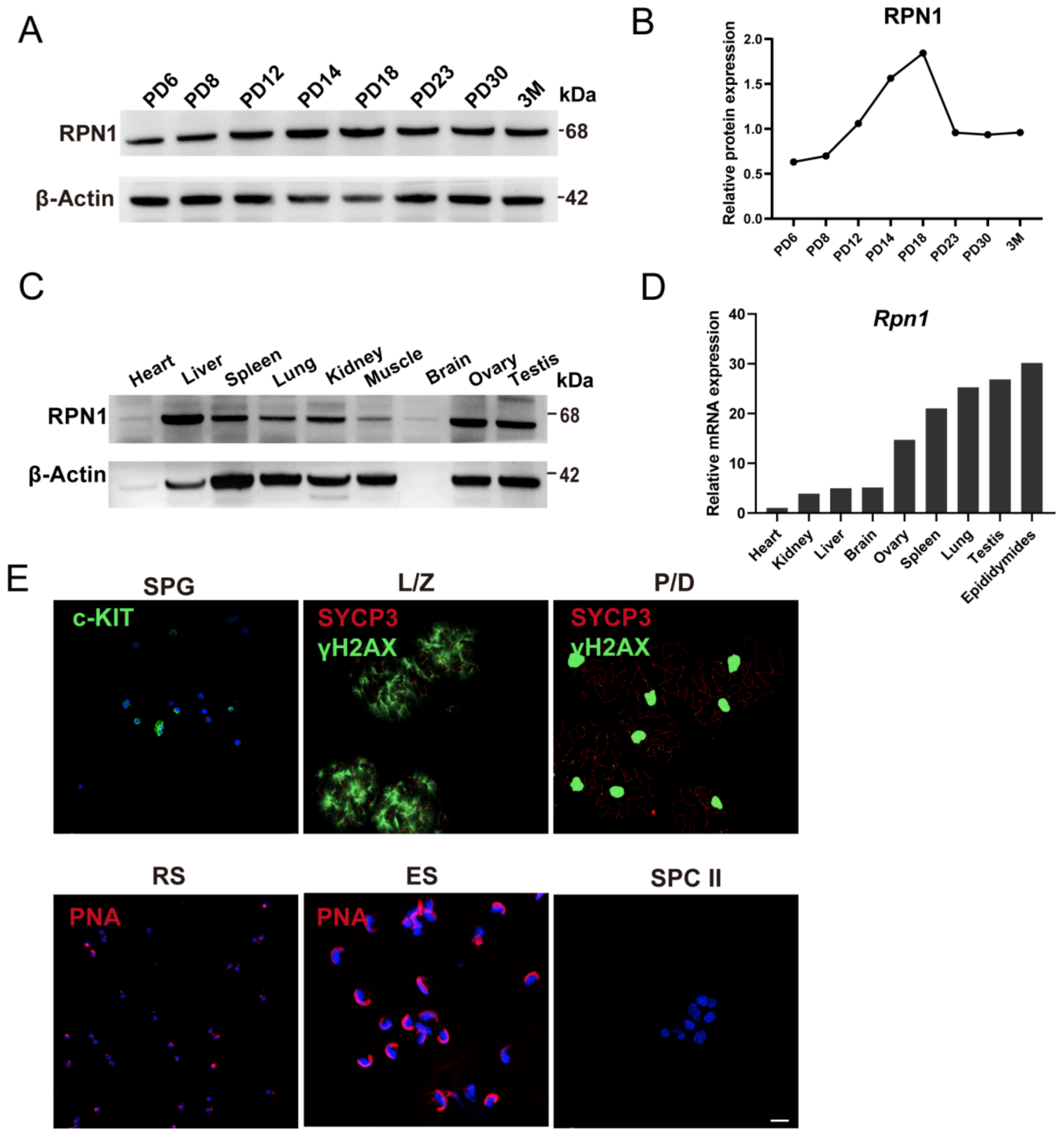


**Figure S1. Expression pattern of RPN1 during spermatogenesis**

**A.** WB detection of RPN1 at different developmental stages in mice testes. β-Actin served as the loading control. **B.** Quantification of the relative expression levels of RPN1 at different ages. The relative expression level of the RPN1 protein was calculated by quantifying the gray value of RPN1/β-Actin for each sample. **C.** WB analysis of RPN1 in various organs of adult mice. β-Actin served as the loading control. **D.** Quantification of the relative expression levels of *Rpn1* at various organs. *Gapdh* served as loading control. **E.** Label and stain the different cells obtained through FACS. Scale bar, 20 μm.

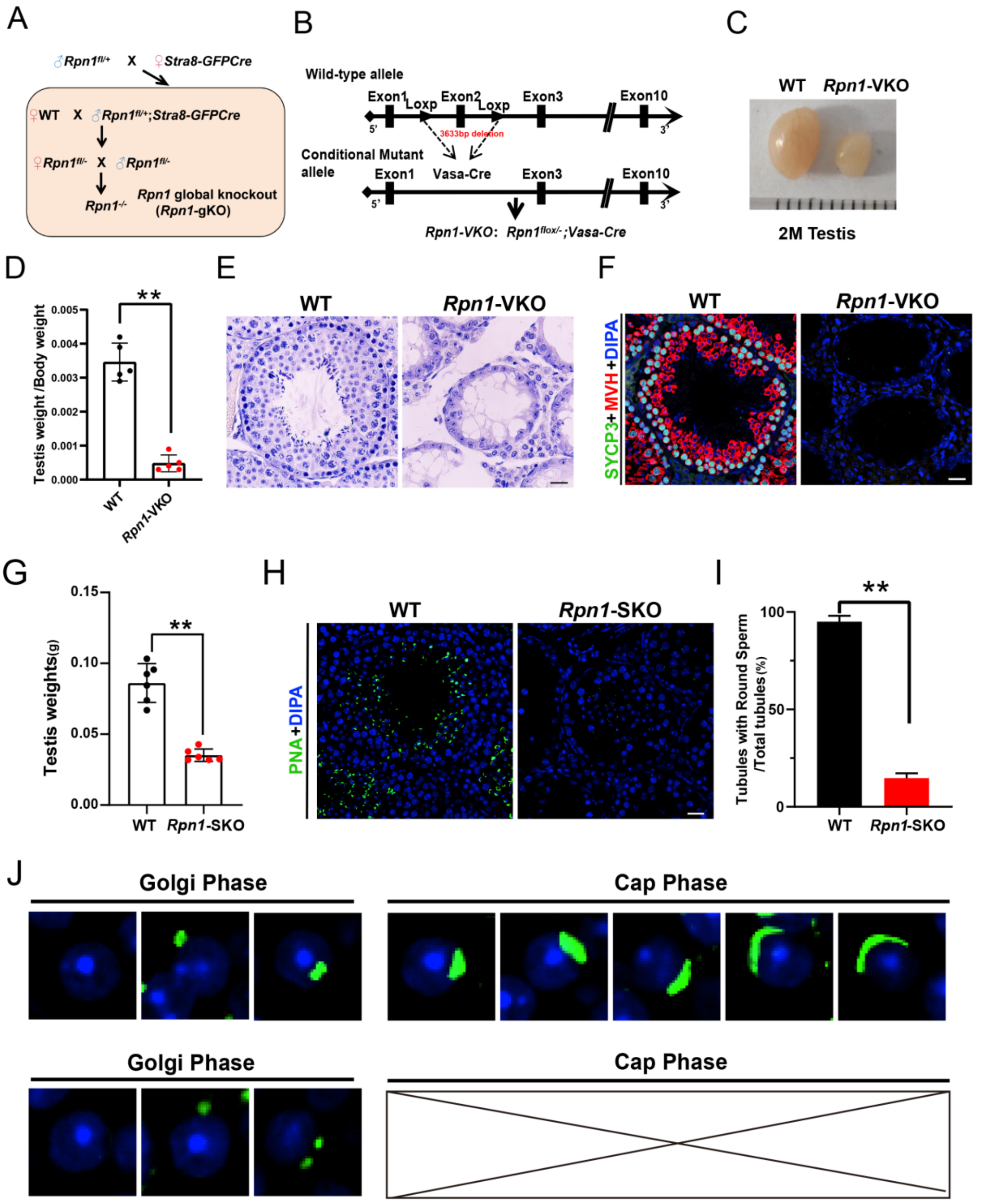


**Figure S2 *Rpn1*-VKO mouse phenotype**

**A.** Schematic diagram of the generation of global *Rpn1* knockout mice. **B.** Schematic of *Rpn1* conditional knockout strategy in germ cells. **C.** Morphology of testes from 2M WT and *Rpn1*-VKO mice. **D.** Testis weight/body weight ratios in WT and *Rpn1*-VKO mice. Data represent means ± SEM. **$p$ < 0.01 by Student's t-test. **E.** Morphological analysis of WT and *Rpn1*-VKO testes by hematoxylin staining. Scale bar, 20 μm. **F.** Immunofluorescence co-staining of SYCP3 (green) and MVH (red) from WT and *Rpn1*-VKO mice. Scale bar, 30 μm. **G.** Testis weight of WT and *Rpn1*-SKO mice. Data

represent means ± SEM. $^{**}p < 0.01$ by Student's t-test. **H.** Analysis of spermiogenesis and acrosome biogenesis in WT and *Rpn1*-SKO mice by fluorescence imaging of spermatids labeled with PNA lectin (green). Scale bar, 30 μm. **I.** Quantification of seminiferous tubules containing round spermatids. **J.** IF staining of PNA (green) in the phases of acrosome biogenesis in WT and *Rpn1*-SKO spermatids. The phases of acrosome biogenesis and corresponding spermiogenesis steps are the Golgi phase (steps 1-3), cap phase (steps 4-7).

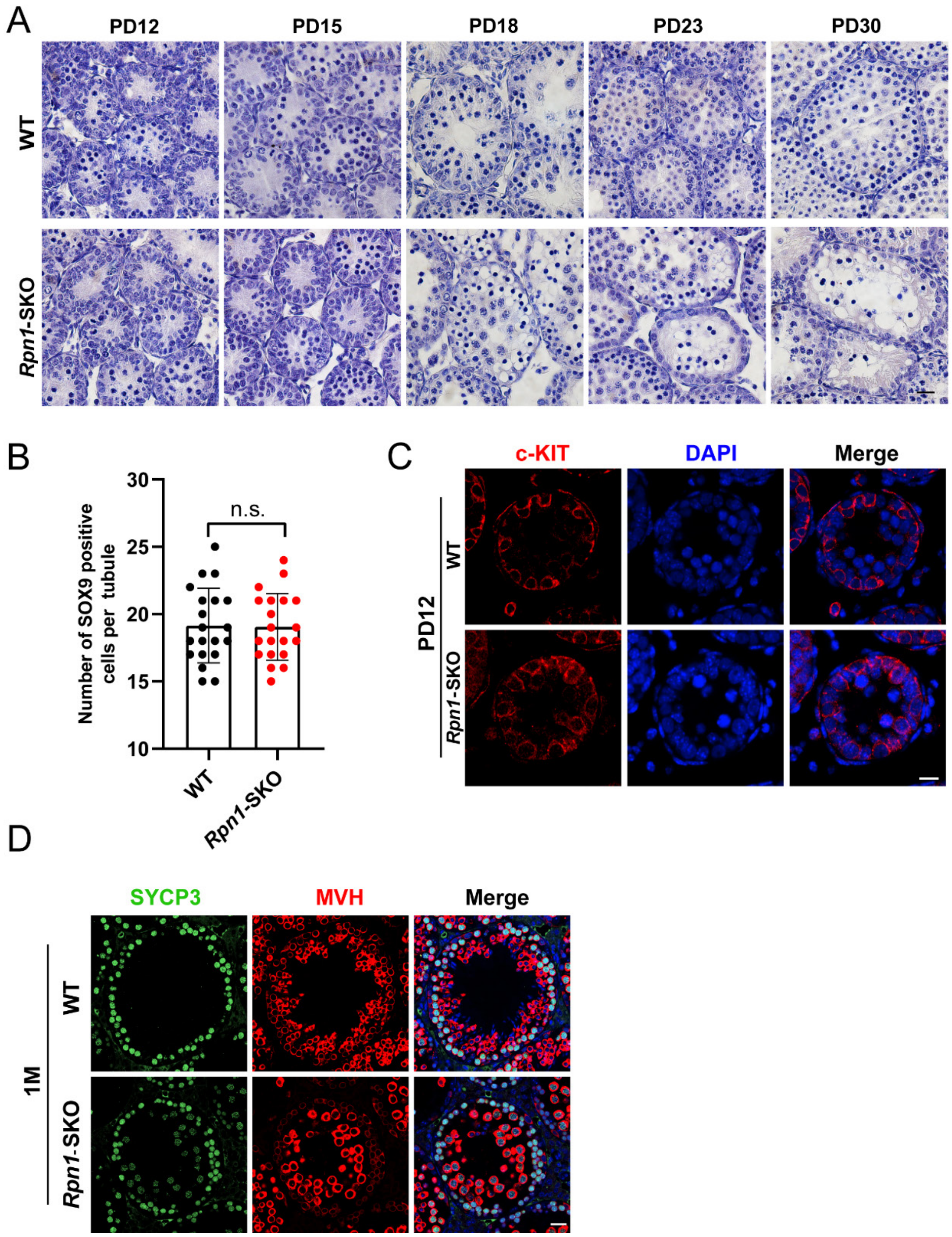


**Figure S3. RPN1 is required for spermatogenesis**

**A.** Hematoxylin staining of WT and *Rpn1*-SKO testes at different ages. Scale bar, 20 μm. **B.** Quantification of $SOX9^{+}$ cells per tubule in PD12 WT and *Rpn1*-SKO mice. Data represent means ± SEM. n.s., not significant. **C.** IF staining for c-KIT (red) in PD12 WT and *Rpn1*-SKO mice. Scale bar, 30 μm. **D.** IF co-staining for SYCP3 (green) and MVH (red) in 1M WT and *Rpn1*-SKO mice. Scale bar, 30 μm.

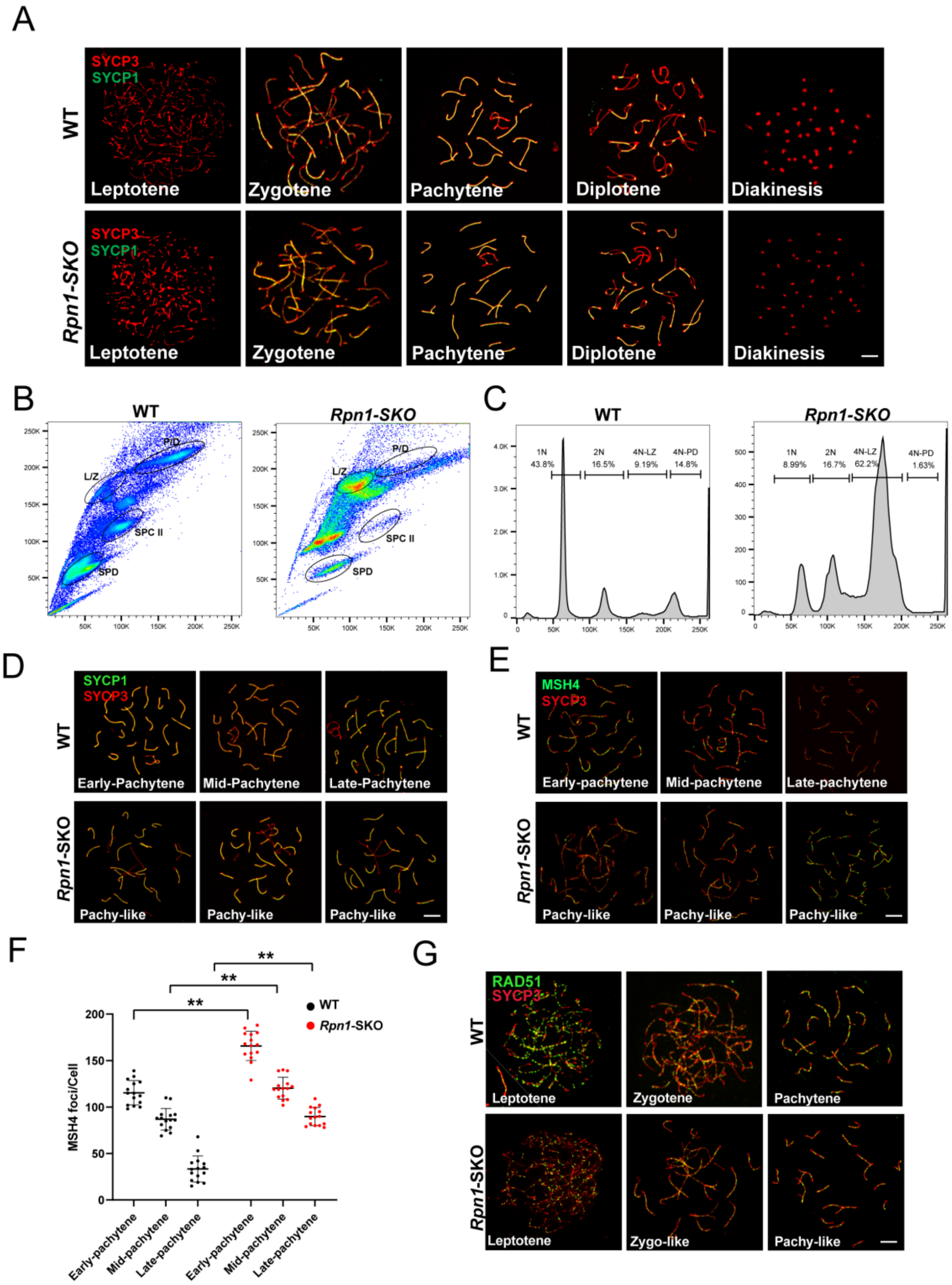


**Figure S4. Abnormal meiosis in spermatocytes with conditional knockout of *Rpn1***

**A.** SYCP3 (red) and SYCP1 (green) co-staining in spermatocyte spreads from 1M WT and *Rpn1*-SKO mice. Scale bar, 5 μm. **B-C.** Representative FACS profiles of distinct spermatogenic populations based on Hoechst fluorescence from adult WT and *Rpn1*-SKO male mice. Histogram peaks correspond to the N, 2N, and 4N cell populations. **D.** Representative images of pachy-like spermatocytes of *Rpn1*-SKO mice. Scale bar, 5 μm. **E.** IF co-staining for SYCP3 (red) and MSH4 (green) in WT and *Rpn1*-SKO mice. Scale bar, 5μm. **F.** Quantification of MSH4 foci per nucleus in WT and *Rpn1*-SKO

mice. Data represent means ± SEM, *$p < 0.05$, **$p < 0.01$ by Student's t-test. **G.** IF co-staining for SYCP3 (red) and RAD51 (green) in WT and *Rpn1*-SKO mice. The dashed line separates the target cell from the adjacent unrelated cell. Scale bar, 5 μm.

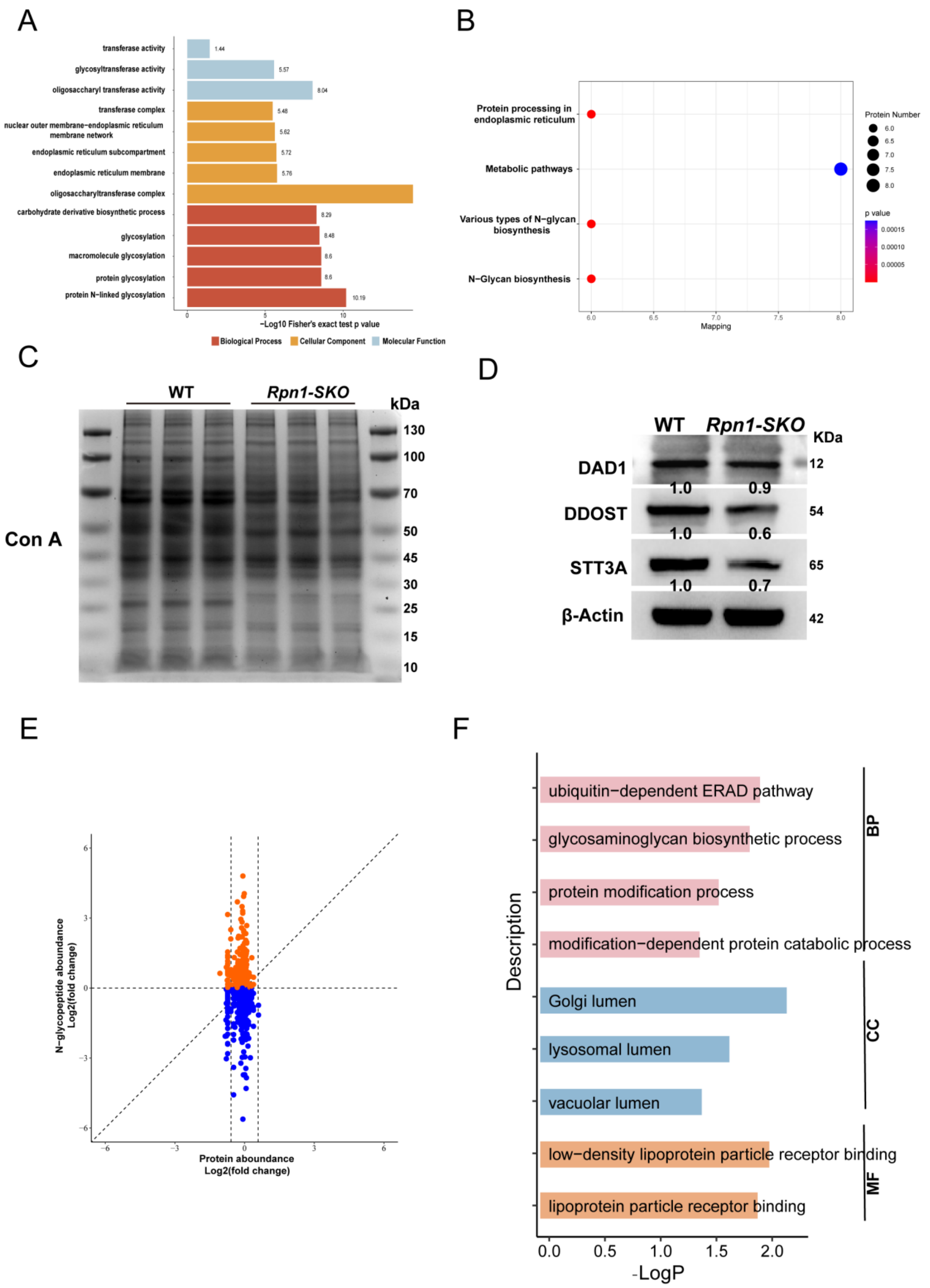


**Figure S5. Proteomics analysis of WT and *Rpn1*-SKO testes**

**A.** GO analysis of significantly downregulated proteins. **B.** The bubble chart demonstrating KEGG pathway enrichment results of downregulated proteins. **C.** WB with Con A lectin in WT and *Rpn1*-SKO testes, staining by Coomassie Brilliant Blue. **D.** The proteomic results were verified by WB, β-Actin served as the loading control.

**E.** Nine-quadrant diagram showing that glycopeptide abundance changes in *Rpn1*-SKO testes are not due to altered protein abundance. **F.** GO analysis of significantly upregulated glycoproteins.

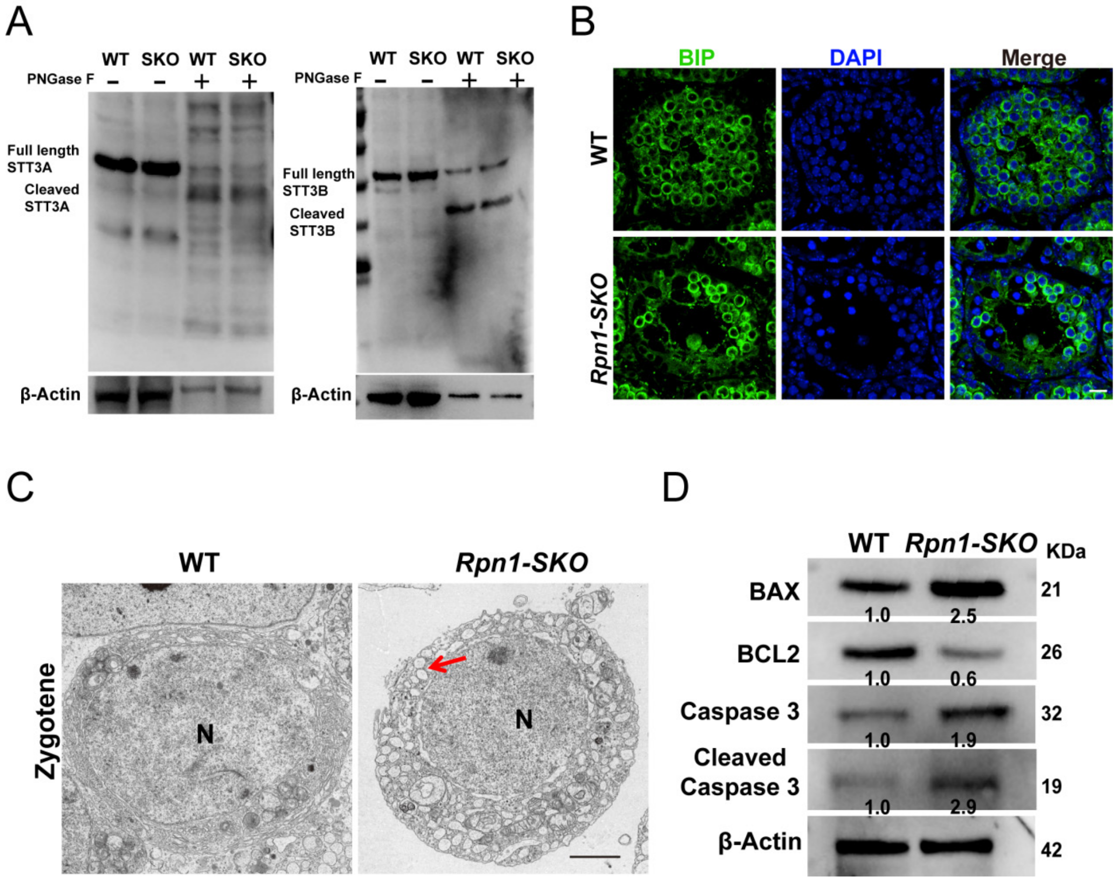


**Figure S6. RPN1 deficiency induces ERS and apoptosis of spermatocytes**

**A.** WB detection of key protein glycosylation levels in adult WT and *Rpn1*-SKO testes. β-Actin served as the loading control. **B.** IF staining for BIP in testes of WT and *Rpn1*-SKO testes. Scale bar, 30 μm. **C.** TEM imaging of ER architecture in testicular sections from 2M WT and *Rpn1*-SKO mice. N, nucleus. Scale bar, 5 μm. **D.** WB detection of apoptosis-related proteins levels in adult WT and *Rpn1*-SKO testes. β-Actin served as the loading control.
